# Predicting panel attrition in longitudinal HRQoL surveys during the COVID-19 pandemic in the US

**DOI:** 10.1186/s12955-022-02015-8

**Published:** 2022-07-06

**Authors:** Tianzhou Yu, Jiafan Chen, Ning Yan Gu, Joel W. Hay, Cynthia L. Gong

**Affiliations:** 1 Department of Pharmaceutical and Health Economics, School of Pharmacy, University of Southern California, Los Angeles, CA USA; 2 School of Nursing and Health Professions, University of San Francisco, San Francisco, CA USA; 3 Schaeffer Center for Health Policy and Economics, School of Pharmacy, University of Southern California, Los Angeles, CA USA; 4 Fetal and Neonatal Institute, Division of Neonatology, Department of Pediatrics, Children’s Hospital Los Angeles, Los Angeles, CA USA

**Keywords:** COVID-19, Panel attrition, Quality of life

## Abstract

**Background:**

Online longitudinal surveys may be subject to potential biases due to sample attrition. This study was designed to identify potential predictors of attrition using a longitudinal panel survey collected during the COVID-19 pandemic.

**Methods:**

Three waves of data were collected using Amazon Mechanical Turk (MTurk), an online crowd-sourced platform. For each wave, the study sample was collected by referencing a US national representative sample distribution of age, gender, and race, based on US census data. Variables included respondents’ demographics, medical history, socioeconomic status, COVID-19 experience, changes of health behavior, productivity, and health-related quality of life (HRQoL). Results were compared to pre-pandemic US norms. Measures that predicted attrition at different times of the pandemic were identified via logistic regression with stepwise selection.

**Results:**

1467 of 2734 wave 1 respondents participated in wave 2 and, 964 of 2454 wave 2 respondents participated in wave 3. Younger age group, Hispanic origin (p ≤ 0.001) and higher self-rated survey difficulty (p ≤ 0.002) consistently predicted attrition in the following wave. COVID-19 experience, employment, productivity, and limited physical activities were commonly observed variables correlated with attrition with specific measures varying by time periods. From wave 1, mental health conditions, average daily hours worked (p = 0.004), and COVID-19 impact on work productivity (p < 0.001) were associated with a higher attrition rate at wave 2, additional to the aforementioned factors. From wave 2, support of social distancing (p = 0.032), being Republican (p < 0.001), and having just enough money to make ends meet (p = 0.003) were associated with predicted attrition at wave 3.

**Conclusions:**

Attrition in this longitudinal panel survey was not random. Besides commonly identified demographic factors that contribute to panel attrition, COVID-19 presented novel opportunities to address sample biases by correlating attrition with additional behavioral and HRQoL factors in a constantly evolving environment. While age, ethnicity, and survey difficulty consistently predicted attrition, other factors, such as COVID-19 experience, changes of employment, productivity, physical health, mental health, and financial situation impacted panel attrition during the pandemic at various degrees.

**Supplementary Information:**

The online version contains supplementary material available at 10.1186/s12955-022-02015-8.

## Background

The coronavirus disease 2019 (COVID-19) has had significant impacts on various aspects of public health. In addition to the clinical consequences resulted from contracting the virus, uninfected individuals are also susceptible to non-clinical consequences resulted from the pandemic such as health care delays and reduced health-related quality of life (HRQoL) [1]. The pressing need for time-sensitive data collection has led to an explosion of research conducted via online platforms, which provide an effective and efficient strategy for researchers to collect data on COVID-19 within a short time period [2–4]. While this methodology may be effective for data collection during the pandemic, it is not without drawbacks [5].

Given the constantly evolving circumstances of the pandemic, longitudinal panels can capture the dynamics of outcomes of interest over time. At the same time, a longitudinal panel may be subject to various types of biases that limit generalizability of the results, especially when conducted online [6]. One such bias is non-response bias, i.e. respondents within the panel not completing follow-up surveys and/or dropping out of the study, which can occur for various, unknown reasons, ranging from loss of interest in the study to an inability to participate due to personal circumstances. Regardless of the reasons, whenever attrition is not random, panel data are subject to potential biases. Consequently, the findings may not be capturing the panel’s true longitudinal changes, and thus leads to threats to sample validity.

Our research team conducted a three-wave longitudinal panel survey online from April 1st 2020 to March 15th 2021 to monitor changes in HRQoL, among other characteristics, throughout the first year in pandemic [1]. This provided a unique opportunity to assess determinants of panel attrition in a longitudinal survey during the pandemic in the US. The objective of the study was to assess the association between an extensive range of survey respondents’ demographic, health behavioral, employment status, HRQoL measures and their participation status in the three waves of the data collection, with the hope of aiding researchers to better interpret future results generated from similar types of panel data, and providing insights on panel data attrition for studies that collect data online during crises such as the COVID-19 pandemic.

## Methods

### Survey overview

We collected a total of three waves of survey data from April 2020 to March 2021 to assess changes in HRQoL over time in the US. Wave 1 data were collected from April 1st to May 6th, 2020 (n = 2734). Wave 2 data was collected from July 4th to September 4th, 2020 (n = 2454). Wave 3 data was collected from January 10th to March 15th, 2021 (n = 2252). We used a panel data structure designed for data collection, i.e., for each wave, while sample attrition occurred, new and additional participants were recruited to ensure that we recruited a comparable sample size for each wave.

### Participant recruitment

We used Amazon’s Mechanical Turk (MTurk) platform to field the survey. Amazon MTurk is an online crowd-sourced platform that allows large-scale surveys to be deployed [7]. Respondents aged 18 years or older and resided in the United States registered as “workers” in MTurk were eligible to participate in our survey. Because the platform is online, all tasks require an active internet connection. There were no other exclusion criteria. The sampling strategy was non-probabilistic as we did not restrict who completed the survey on the platform. Age, gender and race were stratified to be similar to the general US population. Participants were compensated €1.50 (approximately $2 USD) for their time to complete each survey. Informed consent was obtained at the beginning of the survey.

### Measures

We collected information on various sample characteristics including respondents’ demographics, COVID-19 status, HRQoL, health behavior, employment status, and productivity changes related to COVID-19. HRQoL was measured using the EuroQol EQ-5D-5L, the Veterans Rand 12-Item Health Survey (VR-12), Patient Health Questionnaire-4 (PHQ-4), the 2-item Connor-Davidson Resilience Scale (CD-RISC 2), Primary Care PTSD Screen for DSM-5 (PC-PTSD-5), as well as questions selected from the Patient-Reported Outcomes Measurement Information System (PROMIS), and Behavioral Risk Factor Surveillance System (BRFSS) questionnaire. Additional measures were calculated using information collected from the survey (Additional file 1: Appendix 1).

The primary analysis focused on identifying predictors of attrition at waves 2 and 3, referencing the sample characteristics in the previous wave. We also assessed any changes in identified predictors of attrition between different periods of the pandemic.

### Statistical analysis

All study variables were pre-processed by removing outliers and imputing missing values. Specifically, outliers were removed for variables that resulted from open-ended questions such as “How many more/less hours do you sleep than before COVID-19?” or questions that were autogenerated by the survey platform to indicate the time used to complete survey. In addition, unrealistic values (e.g., weight of 5 pounds) were also removed. Missing values were imputed based on appropriate distributions with parameters calculated using sample mean and standard deviation. Differences before and after imputation were checked using Kolmogorov-Smirnoff test (Additional file 1: Appendix 1).

Descriptive analyses were performed on all variables of interest from wave 1 and wave 2. Because only participation status from wave 3 was used in the analysis, sample characteristics of wave 3 were not analyzed. We compared the sample characteristics at wave 1 and wave 2 to US population norms [8–13]. We also compared sample characteristics at wave 1 by wave 2 attrition and sample characteristics at wave 2 by wave 3 attrition, using statistical tests appropriate for the distribution of the measure (e.g., t-test, chi-square test). We then employed standard logistic regression with stepwise selection to identify the most parsimonious model that predicted the attrition at wave 2 and wave 3 using characteristics in the previous wave, respectively. Age, gender, race, and ethnicity were fixed in the regression model based on background knowledge. The a priori significance level for variable entry and removal by the stepwise selection was 5%. The stepwise procedure combines forward selection and backward elimination to produce a list of plausible explanatory variables [14]. While stepwise regression has been critiqued with overfitting the model by including nuisance variables, this issue was of a lesser concern since we were primarily interested in themes represented by the selected variables that were associated with attrition. All analyses were conducted using SAS software version 9.4 (SAS Institute, Cary, NC).

## Results

### Sample characteristics

Wave 1 contained 2734 respondents, amongst whom 1467 (53.7%) participated in wave 2. Wave 2 included a total of 2454 respondents, and 964 (39.3%) of them participated in wave 3. In addition, 261 respondents participated in wave 1 and wave 3 but skipped wave 2. A total of 940 respondents participated in all 3 waves of the survey.

Respondents in Wave 1 ranged from age 18 to 82 years, with a mean (SD) age of 42.6 (± 14.3) years, 1365 (49.9%) were female, 1879 (68.7%) were white, 2446 (89.5%) were non-Hispanic, 1683 (61.6%) had a bachelor or higher degree, and 1276 (46.7%) were married. Wave 2 consisted of 2454 respondents ranging in age also from 18 to 82 years, with a mean (SD) age of 40.6 (± 13.3) years, 1073 (43.7%) were female, 1878 (76.5%) were white, 1987 (81.0%) were non-Hispanic, 1756 (71.5%) had a Bachelor or higher degree, and 1424 (58.0%) were married. Compared to the general US population, our wave 1 sample was slightly older, more likely to be single, and had higher education level. Less individuals identified as Hispanic or Black but more identified as multi-race. Income was more bell-shaped than the general US population. Wave 2 participants were younger, less likely to be female, and more likely to be married compared to the general US population; on the other hand, the race and ethnicity composition was comparable (Table 1).

**Table 1:** Sample demographic characteristics vs. US population

	Wave 1 sample, n (%) n = 2734	Wave 2 sample, n (%) n = 2454	US population (%)	References
Age, years, median	39.0	37.0	38.3	US Census Bureau
Age, years, mean (SD)	42.6 (14.3)	40.6 (13.3)		
*Age group, n (%)*				
18–24	192 (7.0)	101 (4.1)	10.3	
25–34	810 (29.6)	911 (37.1)	14.0	
35–44	660 (24.1)	596 (24.3)	12.6	
45–54	368 (13.5)	380 (15.5)	12.6	
55–64	456 (16.7)	299 (12.2)	12.8	
≥ 65	248 (9.1)	167 (6.8)	16.3	
*Gender, n (%)*				US Census Bureau
Male	1340 (49.0)	1371 (55.9)	49.0	
Female	1365 (49.9)	1073 (43.7)	51.0	
Other	29 (1.1)	10 (0.4)	–	
*Race, n (%)*				US Census Bureau
White	1879 (68.7)	1878 (76.5)	76.3	
American Indian or Alaska Native	17 (0.6)	24 (1.0)	1.3	
Asian	184 (6.7)	145 (5.9)	5.9	
Black or African American	198 (7.2)	307 (12.5)	13.4	
Native Hawaiian or Other Pacific Islander	4 (0.2)	1 (0.0)	0.2	
Multiple races	408 (14.9)	65 (2.7)	2.8	
Other	44 (1.6)	34 (1.4)	–	
*Ethnicity, n (%)*				US Census Bureau
Non-Hispanic	2446 (89.5)	1987 (81.0)	81.5	
Hispanic	268 (9.8)	440 (17.9)	18.5	
Prefer not to say	20 (0.7)	27 (1.1)	–	
*Education, n (%)*				US Census Bureau
Less than high school degree	14 (0.5)	7 (0.3)	10.6	
High school degree or equivalent (eg. GED)	264 (9.7)	178 (7.3)	28.3	
Some college but no degree	457 (16.7)	275 (11.2)	18.0	
Associate degree	316 (11.6)	237 (9.7)	9.8	
Bachelor’s degree	1203 (44.0)	1203 (49.0)	21.3	
Graduate degree	480 (17.6)	553 (22.5)	12.0	
Don’t know	–	1 (0.0)	–	
*Marital status, n (%)*				US Census Bureau
Single	1070 (39.1)	785 (32.0)	33.8	
Married	1276 (46.7)	1424 (58.0)	47.8	
Separated	23 (0.8)	29 (1.2)	1.9	
Divorced	265 (9.7)	170 (6.9)	10.9	
Widowed	75 (2.7)	35 (1.4)	5.7	
Prefer not to say	25 (0.9)	11 (0.5)	–	
*Region, n (%)*				US Census Bureau
Northeast	498 (18.2)	440 (17.9)	17.1	
Midwest	525 (19.2)	425 (17.3)	20.8	
South	1004 (36.7)	899 (36.6)	38.3	
West	707 (25.9)	690 (28.1)	23.9	
*Income, n (%)*				US Census Bureau
Less than $20,000	281 (10.3)	209 (8.5)	13.1	
$20,000–$34,999	423 (15.5)	351 (14.3)	12.3	
$35,000–$49,999	479 (17.5)	482 (19.6)	11.7	
$50,000–$74,999	688 (25.2)	702 (28.6)	16.5	
$75,000–$99,999	440 (16.1)	429 (17.5)	12.3	
$100,000–$149,999	306 (11.2)	200 (8.2)	15.5	
Over $150,000	117 (4.3)	81 (3.3)	18.5	

*SD* standard deviation

^*^Percentages may not add up to 100% due to rounding

Before any adjustment for confounding, age and ethnicity in the wave 1 sample were significantly different by wave 2 attrition. Those who were younger, of Hispanic origin were more likely to drop out in wave 2. Age, race, ethnicity, education, marital status, region, income, and political affiliation in the wave 2 sample were all significantly different by wave 3 attrition. Wave 2 participants who were younger, black, Hispanic, married, college-educated, Republican, lived in the west, and had an income between $35,000–$74,999 were more likely to drop out at wave 3 (Table 2). Other variables that were significantly different by wave 2 and wave 3 attrition are shown in the appendices (Additional file 1: Appendices 2 and 3).

**Table 2:** Demographic characteristics by attrition

	Wave 1 characteristics			Wave 2 characteristics		
	Not in wave 2 (n = 1267)	In wave 2 (n = 1467)	P value	Not in wave 3 (n = 1490)	In wave 3 (n = 964)	P value
Age, years, mean (SD)	41.6 (14.7)	43.4 (14.0)	0.002*	37.7 (11.9)	45.3 (14.0)	< 0.001*
*Age group, n (%)*			< 0.001*			< 0.001*
18–24	118 (9.3)	74 (5.0)		71 (4.8)	30 (3.1)	
25–34	398 (31.4)	412 (28.1)		679 (45.6)	232 (24.1)	
35–44	287 (22.7)	373 (25.4)		346 (23.2)	250 (25.9)	
45–54	141 (11.1)	227 (15.5)		216 (14.5)	164 (17.0)	
55–64	217 (17.1)	239 (16.3)		129 (8.7)	170 (17.6)	
> = 65	106 (8.4)	142 (9.7)		49 (3.3)	118 (12.2)	
*Gender, n (%)*			0.808			0.343
Male	613 (48.4)	727 (49.6)		850 (57.0)	521 (54.0)	
Female	641 (50.6)	724 (49.4)		634 (42.6)	439 (45.5)	
Other	13 (1.0)	16 (1.1)		6 (0.4)	4 (0.4)	
*Race, n (%)*			0.129			< 0.001*
White	851 (67.2)	1028 (70.1)		1126 (75.6)	752 (78.0)	
Black or African American	92 (7.3)	106 (7.2)		235 (15.8)	72 (7.5)	
Asian	79 (6.2)	105 (7.2)		59 (4.0)	86 (8.9)	
American Indian or Alaska Native	10 (0.8)	7 (0.5)		19 (1.3)	5 (0.5)	
Native Hawaiian or Other Pacific Islander	3 (0.2)	1 (0.1)		0 (0.0)	1 (0.1)	
Multiple races	213 (16.8)	195 (13.3)		35 (2.3)	30 (3.1)	
Other	19 (1.5)	25 (1.7)		16 (1.1)	18 (1.9)	
*Ethnicity, n (%)*			< 0.001*			< 0.001*
Non-Hispanic	1086 (85.7)	1360 (92.7)		1071 (71.9)	916 (95.0)	
Hispanic	170 (13.4)	98 (6.7)		397 (26.6)	43 (4.5)	
Prefer not to say	11 (0.9)	9 (0.6)		22 (1.5)	5 (0.5)	
*Education, n (%)*			0.130			< 0.001*
Less than high school degree	5 (0.4)	9 (0.6)		3 (0.2)	4 (0.4)	
High school degree or equivalent (eg. GED)	114 (9.0)	150 (10.2)		78 (5.2)	100 (10.4)	
Some college but no degree	210 (16.6)	247 (16.8)		125 (8.4)	150 (15.6)	
Associate degree	128 (10.1)	188 (12.8)		113 (7.6)	124 (12.9)	
Bachelor’s degree	584 (46.1)	619 (42.2)		793 (53.2)	410 (42.5)	
Graduate degree	226 (17.8)	254 (17.3)		377 (25.3)	176 (18.3)	
Don’t know	0 (0.0)	0 (0.0)		1 (0.1)	0 (0.0)	
*Marital status, n (%)*			0.348			< 0.001*
Single	483 (38.1)	587 (40.0)		403 (27.0)	382 (39.6)	
Married	610 (48.1)	666 (45.4)		1013 (68.0)	411 (42.6)	
Separated	7 (0.6)	16 (1.1)		16 (1.1)	13 (1.3)	
Divorced	117 (9.2)	148 (10.1)		48 (3.2)	122 (12.7)	
Widowed	36 (2.8)	39 (2.7)		7 (0.5)	28 (2.9)	
Prefer not to say	14 (1.1)	11 (0.7)		3 (0.2)	8 (0.8)	
*Region, n (%)*			0.132			< 0.001*
Northeast	237 (18.7)	261 (17.8)		275 (18.5)	165 (17.1)	
Midwest	250 (19.7)	275 (18.7)		230 (15.4)	195 (20.2)	
South	436 (34.4)	568 (38.7)		529 (35.5)	370 (38.4)	
West	344 (27.1)	363 (24.7)		456 (30.6)	234 (24.3)	
*Income, n (%)*			0.264			< 0.001*
Less than $20,000	131 (10.3)	150 (10.2)		109 (7.3)	100 (10.4)	
$20,000–$34,999	201 (15.9)	222 (15.1)		196 (13.2)	155 (16.1)	
$35,000–$49,999	242 (19.1)	237 (16.2)		334 (22.4)	148 (15.4)	
$50,000–$74,999	313 (24.7)	375 (25.6)		465 (31.2)	237 (24.6)	
$75,000–$99,999	184 (14.5)	256 (17.5)		260 (17.4)	169 (17.5)	
$100,000–$149,999	144 (11.4)	162 (11.0)		86 (5.8)	114 (11.8)	
Over $150,000	52 (4.1)	65 (4.4)		40 (2.7)	41 (4.3)	
*Insurance status, n (%)*			0.099			< 0.001*
Commercial or private	578 (45.6)	726 (49.5)		419 (28.1)	494 (51.2)	
Medicare	192 (15.2)	186 (12.7)		593 (39.8)	154 (16.0)	
Medicaid/ACA	244 (19.3)	269 (18.3)		256 (17.2)	194 (20.1)	
Self-pay/None/Don’t know	253 (20.0)	286 (19.5)		222 (14.9)	122 (12.7)	
*Political affiliation*			0.858			< 0.001*
Republican	373 (29.4)	421 (28.7)		589 (39.5)	248 (25.7)	
Democrat	596 (47.0)	681 (46.4)		596 (40.0)	451 (46.8)	
Independent	288 (22.7)	354 (24.1)		278 (18.7)	225 (23.3)	
None of the above	10 (0.8)	11 (0.7)		27 (1.8)	40 (4.1)	

*SD* standard deviation

^*^Significance at 0.05 level

### Wave 1 predictors of attrition in wave 2

Table 3 presents odds ratios calculated based on results from the logistic regression after stepwise selection. After controlling for all other measures collected in the wave 1 survey, age, race, ethnicity, experiencing COVID-19-like symptoms, change of normal diet, average hours of sleep per day, hours missed from work due to COVID-19, COVID-19 impact on productivity, self-rated survey difficulty, and specific HRQoL questions from the VR-12 (Q2a, “Does your health now limit you in moderate activities, such as moving a table, pushing a vacuum cleaner, bowling or playing golf? If so, how much?”; Q6b, “How much of the time during the past 4 weeks did you have a lot of energy?”; Q9, “Compared to one year ago, how would you rate your emotional problems now?”) were found to be associated with attrition at wave 2.

**Table 3:** Factors associated with panel attrition

Predictor in the previous wave	Attrition at wave 2			Attrition at wave 3		
	Odds ratio	Lower 95% CI	Upper 95% CI	Odds ratio	Lower 95% CI	Upper 95% CI
*Age group*						
18–24	–	–	–	–	–	–
25–34	0.596*	0.423	0.839	0.636	0.375	1.079
35–44	0.553*	0.388	0.786	0.378*	0.219	0.654
45–54	0.426*	0.291	0.625	0.313*	0.175	0.560
55–64	0.735	0.508	1.064	0.241*	0.133	0.437
≥ 65	0.620*	0.409	0.939	0.078*	0.038	0.159
*Gender*						
Male	–	–	–	–	–	–
Female	1.064	0.903	1.255	1.076	0.872	1.328
Other	0.676	0.287	1.590	1.788	0.432	7.406
*Race*						
White	–	–	–	–	–	–
American Indian or Alaska Native	1.259	0.447	3.548	0.673	0.181	2.506
Asian	0.925	0.668	1.281	0.573*	0.381	0.861
Black or African American	0.906	0.660	1.244	0.907	0.618	1.332
Multiple races	1.630*	1.297	2.048	0.653	0.351	1.217
Native Hawaiian or Other Pacific Islander	2.674	0.254	28.129	< .001	< .001	> 999.999
Other/prefer not to say	0.717	0.359	1.432	0.437	0.164	1.160
*Ethnicity*						
Non-Hispanic	–	–	–	–	–	–
Hispanic	1.698*	1.266	2.279	2.097*	1.344	3.273
Prefer not to say	2.250	0.798	6.350	4.060	0.952	17.313
*Marital Status*						
Married	–	–	–	–	–	–
Single	–	–	–	0.774*	0.600	0.999
Widowed	–	–	–	0.505	0.205	1.242
Divorced	–	–	–	0.509*	0.338	0.767
Separated	–	–	–	0.567	0.206	1.565
Prefer not to say	–	–	–	0.215	0.045	1.039
*Insurance*						
Commercial or private	–	–	–	–	–	–
Medicare	–	–	–	2.298*	1.601	3.299
Medicaid/ACA	–	–	–	0.970	0.721	1.303
Self-pay/none/don’t know	–	–	–	1.683*	1.216	2.329
*Political affiliation*						
Democrat	–	–	–	–	–	–
Republican	–	–	–	1.654*	1.262	2.167
Independent	–	–	–	1.237	0.939	1.629
None of the above	–	–	–	0.918	0.514	1.642
*Medical history*						
Arthritis	–	–	–	0.621*	0.413	0.932
Diabetes	–	–	–	1.599*	1.078	2.372
Stroke	–	–	–	3.682*	1.101	12.308
Bronchitis	–	–	–	0.386*	0.217	0.686
*Smoking*						
Never	–	–	–	–	–	–
In the past	–	–	–	1.756*	1.375	2.242
Currently	–	–	–	1.430*	1.057	1.935
*BMI category*						
Normal weight	–	–	–	–	–	–
Underweight	–	–	–	1.651*	1.076	2.534
Overweight	–	–	–	0.836	0.652	1.071
Obesity	–	–	–	1.059	0.786	1.428
Medical care deferred due to COVID-19	–	–	–	1.612*	1.238	2.101
Diagnosed with COVID-19	–	–	–	5.026*	2.026	12.473
Experienced COVID-19-like symptoms not serious enough to require hospitalization	1.384*	1.052	1.821	–	–	–
Support social distancing policy	–	–	–	1.047*	1.004	1.092
Change of normal diet	1.246*	1.042	1.490	–	–	–
Average hours of sleep per day (1–15 scale)	1.103*	1.046	1.162	–	–	–
*Employment change*						
No change	–	–	–	–	–	–
Work from home	–	–	–	1.327*	1.067	1.651
Lost job	–	–	–	2.028*	1.035	3.975
Laid off temporarily	–	–	–	0.577*	0.337	0.988
*Work deemed essential*						
No	–	–	–	–	–	–
Yes	1.223*	1.020	1.466	–	–	–
Don’t know	1.481	0.869	2.524	–	–	–
Average hours worked per day (0–12 scale)	0.966*	0.944	0.989	–	–	–
COVID-19 impact on productivity (0–10 scale)	1.061*	1.028	1.095	–	–	–
*BRFSS, Finances by the end of the month*						
End up with some money left over	–	–	–	–	–	–
Just enough money to make ends meet	–	–	–	1.435*	1.132	1.821
Not enough money to make ends meet	–	–	–	1.394	0.957	2.031
Don't know / Not sure	–	–	–	1.381	0.582	3.279
Prefer not to answer	–	–	–	2.738	0.877	8.543
EQ-5D-5L VAS Score	–	–	–	1.011*	1.005	1.017
*VR-12 Q2a, Moderate activity*						
Not limited at all	–	–	–	–	–	–
Limited a little	1.355*	1.088	1.688	–	–	–
Limited a lot	0.885	0.608	1.288	–	–	–
*VR-12 Q3b, Limited in the kind of work or activities due to physical health*						
None of the time	–	–	–	–	–	–
Some of the time	–	–	–	2.234*	1.524	3.276
A little of the time	–	–	–	1.502*	1.107	2.039
Most of the time	–	–	–	2.221*	1.367	3.609
All of the time	–	–	–	1.404	0.748	2.636
*VR-12 Q6b, Have a lot of energy*						
None of the time	–	–	–	–	–	–
A little of the time	1.436*	1.036	1.989	–	–	–
Some of the time	1.778*	1.289	2.450	–	–	–
A good bit of the time	1.202	0.861	1.676	–	–	–
Most of the time	1.295	0.913	1.836	–	–	–
All of the time	1.337	0.824	2.170	–	–	–
*VR-12 Q9, Emotional problems compared to 1 year ago*						
About the same	–	–	–	–	–	–
Slightly better	1.169	0.921	1.483	–	–	–
Much better	1.974*	1.424	2.736	–	–	–
Slightly worse	0.950	0.773	1.166	–	–	–
Much worse	0.951	0.672	1.345	–	–	–
Self-rated survey difficulty (0–10 scale)	1.075*	1.026	1.126	1.125*	1.061	1.193

*CI* confidence interval

^*^Significant at 0.05 level

Those aged 18–24 were significantly more likely to drop out of the study at wave 2 compared to other age groups. Respondents belonging to multiple race groups (OR 1.630, 95% CI 1.297–2.048) and of Hispanic origin (OR 1.698, 95% CI 1.266, 2.279) were more likely to drop out. Experiencing COVID-19-like symptoms but not requiring hospitalization (OR 1.384, 95% CI 1.052–1.821), change of normal diet (OR 1.246, 95% CI 1.042–1.490), and being essential workers (OR 1.223, 95% CI 1.020–1.466) were significantly associated with attrition. Average hours of sleep per day (OR 1.103, 95% CI 1.046–1.162), self-reported COVID-19 impact on productivity (OR 1.061, 95% CI 1.028–1.095), and self-reported survey difficulty (OR 1.075, 95% CI 1.026–1.126) were also positively correlated with attrition, but the association with average hours worked per day (OR 0.966, 95% CI 0.944–0.989) was negative.

As for HRQoL measures, those who were limited in moderate activities were 1.355 (95% CI 1.088, 1.688) times more likely to drop out than those who were not limited at all. Compared to those who reported never having a lot of energy, those who reported having energy “a little of the time” (OR 1.436, 95% CI 1.036–1.989) and “some of the time” (OR 1.778, 95% CI 1.289–2.450) were more likely to drop out, but those who answered “A good bit of the time”, “Most of the time”, or “All of the time” were not. Compared to those who reported no change to their emotional problems, those who felt much better than one year ago were 1.974 (95% CI 1.424, 2.736) times were more likely to drop out.

### Wave 2 predictors of attrition in wave 3

After controlling for all other measures at wave 2, age, race, ethnicity, marital status, insurance type, political affiliation, medical history (arthritis, diabetes, stroke, and bronchitis), smoking history, BMI category, having medical care deferred, diagnosed of COVID-19, supporting social distancing policy, employment change, finances by the end of the month, self-rated survey difficulty, and two HRQoL measures, EQ-5D-5L visual analog scale (VAS) score and VR-12 Q3b (“During the past 4 weeks, were you limited in the kind of work or activities as a result of your physical health?”) were associated with attrition in wave 3.

Those who were younger than 35 years of age were significantly more likely to drop out at wave 3. Asians (OR 0.573, 95% CI 0.381–0.861) were less likely to drop out compared to Whites. Hispanics were 2.097 (95% CI 1.344, 3.273) times as likely to drop out than non-Hispanics. Compared to married respondents, those who were single (OR 0.774, 95% CI 0.600–0.999) and divorced (OR 0.509, 95% CI 0.338–0.767) were less likely to drop out. Those who had insurance from Medicare (OR 2.298, 95% CI 1.601–3.299) and had no insurance (OR 1.683, 95% CI 1.216–2.329) were more likely to drop out than those who had commercial insurance. Compared to Democrats, Republicans were 1.654 times as likely to drop out (95% CI 1.262–2.167). Having a history of arthritis (OR 0.621, 95% CI 0.413–0.932) or bronchitis (OR 0.386, 95% CI 0.217–0.686) was negatively associated with attrition while the association was positive with history of diabetes (OR 1.599, 95% CI 1.078–2.372) and stroke (OR 3.682, 95% CI 1.101–12.308). Those who smoked in the past and who were current smokers were both more likely to drop out than non-smokers. Those who were underweight were also more likely to drop out than people with normal weight (OR 1.651, 95% CI 1.076–2.534).

Diagnosis of COVID-19 (OR 5.026, 95% CI 2.026–12.473), having medical care deferred due to COVID-19 (OR 1.612, 95% CI 1.238–2.101), and supporting the social distancing policy (OR 1.047, 95% CI 1.004–1.092) were all positively correlated with attrition at wave 3. Compared to those who experienced no change to their employment, those who could work from home (OR 1.327, 95% CI 1.067–1.651) and those who lost their jobs (OR 2.028, 95% CI 1.035–3.975) were more likely to drop out, but those who were laid off temporarily were less likely to drop out (OR 0.577, 95% CI 0.337–0.988). Self-rated survey difficulty (OR 1.125, 95% CI 1.061–1.193) was also positively correlated with attrition.

Financial situation by the end of the month in wave 2 was found to be a significant predicter of attrition at wave 3. Specifically, those who had just enough money to make ends meet (OR 1.435, 95% CI 1.132–1.821) were more likely to discontinue participation compared to those who ended the month with some money left over. Overall HRQoL measured by EQ-5D-5L visual analog scale (VAS) score was positively correlated with attrition (OR 1.011, 95% CI 1.005–1.017). Additionally, compared to those who were not at all limited in the kind of work or activities due to physical health, those who were limited to some extents were all more likely to drop out, except for those who were always limited.

## Discussion

In this study, we aimed to compare factors associated with attrition at different times during the pandemic. To our knowledge, this is also the first study that assesses longitudinal panel attrition regarding COVID-19 related health behavioral changes and HRQoL measures during the pandemic in the US. Our response rate from wave 1 to wave 2 was comparable to those reported in another panel attrition study during a similar period of the COVID-19 pandemic [15]. While we enrolled new participants at wave 2, the response rate from wave 2 to wave 3 was lower. This may result from the longer gap between wave 2 and wave 3 but may also suggest a decreased interest in COVID-19 survey participation as the initial shock wore off.

Despite the constantly changing COVID-19 circumstances during the study period, three characteristics consistently predicted attrition. In both wave 1 and wave 2, young adults were more likely to drop out of the study. This is consistent with reports of higher attrition rates with the younger population in the literature [15–19]. Hispanic participants were also more likely to drop out in both of our samples. Furthermore, self-rated survey difficulty was positively associated with attrition. One study reported that experience with past surveys could be predictive of panel attrition [20]. Participants with an initial negative survey experience are less likely to continue participation. Therefore, to reduce attrition, longitudinal survey designers should balance questionnaire granularity with respondent burden.

While race has also consistently been a predictor of attrition in our data, the specific association remains elusive. The literature is also inconsistent on the effect of race groups on attrition. Studies have found higher dropout rates for Blacks, Asians, American Indians or Alaska Natives, and multiple race groups in various settings but another study reported no such relationship [15, 16, 18, 21, 22]. Although unclear from our data, it is possible that the change in the specific association between race group and attrition during different time periods results from heterogeneous COVID-19 shock on different race groups. This would also explain why evidence from literature is not consistent as each study was reporting an effect specific to the survey topic and study setting.

We found that self-reported concerns for COVID-19 were associated with attrition in both time periods but was expressed by experience of COVID-19 symptoms from wave 1 to wave 2 and confirmed COVID-19 diagnosis from wave 2 to wave 3, suggesting a shift of attention that coincided with the spiking number of new COVID-19 cases in the US in the latter time period [23]. Similarly, employment measures were also predictive of attrition in both time periods but was represented by essential worker status and employment change, respectively, which indicates a shift of job-related concerns. Health behavioral and work productivity changes were only significant in the first half of the study when people were still adjusting to the new lifestyle with capricious lockdown and social distancing policies. Repeatedly, we observe the pattern that while several factors are persistently associated with attrition, the selected measures adapt to reflect the most concerning matter at the time. This dynamic nature calls for researchers’ attention to the circumstance of the data collection, in addition to widely recognized drivers of attrition in the literature.

We found that higher EQ-5D-5L VAS score was predictive of the attrition at wave 3. This result differs from the literature in which one study reported higher participation at multiple time points among cancer survivors with higher HRQoL scores measured by the European Organization for the Research and Treatment of Cancer Quality of Life Questionnaire (EORTC QLQ-C30) and another study reported no significant association between survey participation and subjective well-being rated on a 0–10 scale [24, 25]. One possible explanation for this counter-intuitive relationship is that the EQ-5D-5L VAS score may be capturing some unidentified aspect of HRQoL that negatively affected the survey participation. For example, respondents who had higher hopes and were more optimistic about the pandemic might be more dismissive of the survey. Despite the elusive mechanism, this presents a net effect of HRQoL on survey participation, incorporating not only identified but also unidentified aspects of HRQoL such as resilience. One suggestive piece of evidence is the significant difference between those who lost their jobs and those who were laid off temporarily. While financial stress exists in both circumstances, those who were laid off temporarily expected a re-employment in the future and were more resilient about the situation. Another piece of evidence is that measures of ideology such as political affiliation and support of social distancing policy became predictive of attrition in wave 3 but not in wave 2.

Remaining results from the HRQoL surveys suggest that limited physical activity is predictive of attrition in both time periods, despite being represented by different HRQoL questions. Mental health only predicts panel attrition between wave 1 and wave 2. This is consistent with the observation that health behavioral and productivity changes was predictive in and only in that same time period. However, it is inconclusive from these findings how mental health status specifically impacts panel attrition. A study focused on mental health found that those who completed only one or two surveys had higher baseline prevalence of anxiety/depression symptoms than those who completed all four surveys [15]. In our study, while there were significant differences in anxiety/depression measured by EQ-5D-5L and by a few VR-12 questions before controlling for other variables at wave 1 (Additional file 1: Appendix 2), we did not see a similar trend after adjustment. Overall, the impact of HRQoL on panel attrition is found to be bell-shaped. This may result from both low numbers of observations for participants at the extreme (Additional file 1: Appendices 2 and 3) and lower stability for participants in the middle to remain unchanged. The latter explanation is supported by the observation that improvement of emotional health compared to one year ago was significantly associated with attrition. This suggests that change in HRQoL, rather than the specific level of HRQoL, affects attrition.

Longitudinal HRQoL assessment is vital to healthcare research during the current pandemic and online surveys will continue to be a major data source for such research. However, restricting analyses to those who always respond may lead to biased results. Our data reveal some patterns of panel attrition during the COVID-19 pandemic based on demographic, behavioral, and HRQoL characteristics of the survey participants. Furthermore, we identify three categories for predictors of panel attrition, including 1) predictors that affect attrition consistently regardless of survey topic and circumstances of the study (e.g., age and survey difficulty), 2) common themes that are consistently associated with attrition but the specific predictor and association may vary given the circumstances (e.g., race and employment), and 3) predictors or themes that are context-specific (e.g., behavioral and productivity changes). Because both common themes and context-specific predictors can lead to heterogeneous attrition, longitudinal panel studies should always attempt to assess attrition bias before drawing any conclusions.

The current pandemic created an unnatural and constantly evolving situation that is substantially different from pre-pandemic life (e.g., work-from-home orders, movement restrictions, online learning, etc.), thus making it challenging to compare pre-pandemic and pandemic outcomes without violations of the “Ceteris paribus” principle. Our findings may be, to some extent, specific to the COVID-19 pandemic. For example, most non-essential workers were spending much more time at home/online than before the pandemic. However, given that MTurk is a platform for which respondents voluntarily participate, we believe that our conclusions are still relevant to the post-pandemic era. For example, future studies may want to consider more variables when trying to minimize attrition rates in the design stage, or to understand the effects of attrition on results interpretation. While attrition is often not random, most patterns may be predictable based on prior knowledge. Our analysis produced a partially context-specific explanation as to how attrition manifested. Future research can examine our findings and use our categorization of attrition predictors to infer factors that would contribute to attrition in a different context. Additional measures may also be taken to improve follow-up rates and ensure sufficient data collection in the targeted population (e.g., providing compensation in gift cards from Amazon or coffee shop instead of grocery stores to attract younger population), allowing for better control of attrition in future longitudinal studies, although this must be balanced against ensuring sample representativeness. Ultimately, researchers should be aware of and at minimum, acknowledge non-random attrition when interpreting their results.

### Limitations

This study has several limitations. First, we recruited participants from the MTurk platform. MTurk has been shown to have mixed external validity to the general US population [26–35]. Second, our sample was stratified on age, gender and race. Additional measures not listed could improve the generalizability of our sample. Third, our wave 2 sample included participants from wave 1. These participants were more likely to respond to additional surveys, given that they already completed one round of follow-up. Fourth, the gap between wave 2 and wave 3 was longer than that between wave 1 and wave 2, which could lead to increased attrition rates. Finally, the survey questions remained virtually unchanged in all 3 waves, perhaps contributing to respondent burden and leading to higher rates of attrition.

## Conclusions

Previous research on panel attrition focused on demographic measures and personality traits. However, the societal disruption caused by the COVID-19 pandemic presents new challenges for follow-up in longitudinal panels. We identified multiple demographics, behavioral, and HRQoL measures that predicted attrition in our panel. These results suggest the need to refresh existing considerations when conducting longitudinal panel surveys during the COVID-19 pandemic. Future research may use our findings to improve study design and data interpretation.

## Supplementary Information

**Additional file 1**. Appendices.

## Data Availability

The datasets used and analyzed during the current study are available from the corresponding author on reasonable request.

## Abbreviations

BRFSS: Behavioral Risk Factor Surveillance System
CD-RISC 2: The 2-item Connor-Davidson Resilience Scale
CI: Confidence interval
COVID-19: Coronavirus disease 2019
EORTC QLQ-C30: European Organization for the Research and Treatment of Cancer Quality of Life Questionnaire (version 3)
EQ-5D-5L: EuroQol-5D-5L
HRQoL: Health-related quality of life
MTurk: Mechanical Turk
OR: Odds ratio
PC-PTSD-5: Primary Care PTSD Screen for DSM-5
PHQ-4: Patient Health Questionnaire-4
PROMIS: Patient-Reported Outcomes Measurement Information System
VAS: Visual analog scale
VR-12: Veterans Rand 12-Item Health Survey
