# Supplementary material for: Predicting panel attrition in longitudinal HRQoL surveys during the COVID-19 pandemic in the US
Source: Health Qual Life Outcomes. 2022 Jul 6;20:104. doi: 10.1186/s12955-022-02015-8 (PMC9258760; doi:10.1186/s12955-022-02015-8)
Supplement: Supplementary file 1 — Additional file 1. Appendices. [file 12955_2022_2015_MOESM1_ESM.docx]

**Appendix 1. Survey variables processing**

All variables collected directly from the survey were subject to data processing, including outlier removal and imputation for missing values if necessary. Outliers were removed for responses from measures that had abnormal values (eg. weight of 5 pounds), autogenerated by the survey platform (eg. time used to complete EQ-5D-5L component of the survey), and were from open questions (eg. How many more/less hours do you sleep than before COVID-19?). Values that were larger than the third quartile by 1.5 times of the interquartile range (IQR) or smaller than the first quartile by 1.5 times of the IQR were considered as outliers and removed.

Appropriate distributions were fit to the survey variables to impute for missing values from the survey and from outlier removal. Age, height, and weight were fitted with normal distributions. More/less hours of sleep, more/less spending were fitted with gamma distributions because these variables were right-skewed and left-bounded by 0. EQ-5D-5L VAS score and hours missed from work due to COVID/non-COVID reasons were fitted with a beta distribution because they were left-bounded by 0 and right-bounded by 100 and 99, respectively. Remaining variables were fitted with table distributions. The parameters of distributions were calculated by method of moments after outlier removal. Missing values were imputed by random selection based on the respective distribution. Therefore, by definition, imputation did not alter the distribution and type (numeric/categorical) of the variable. The variable differences before and after data processing were checked using Kolmogorov-Smirnoff test (KS test). Some incidental significant differences (Hours of work missed due to/ not due to COVID-19 and EQ-5D-5L VAS) were observed and examined individually. The pre- and post-imputation cumulative distribution functions (CDF) of these variables were examined and the imputation was determined to be successful.

Secondary composite measures were then calculated, including BMI category, PHQ-4 group, average COVID-19 impact on productivity, EQ-5D-5L utility score, total completion time for the HRQoL component of the survey, and average self-rated HRQoL difficulty and usefulness. These calculations were applied to both pre- and post-imputation data for comparison. Composite measures replaced survey measures in our analysis when applicable to avoid collinearity.

| **Variable collected from wave 1 or wave 2 survey** | **Outlier removal** | **Distribution used for imputation** | **P-value from KS test by wave 2 participation** | **P-value from KS test by wave 3 participation** |
| --- | --- | --- | --- | --- |
| Age | No | Normal | 1.000 | 1.000 |
| Gender | No | Table | 1.000 | 1.000 |
| Race group | No | Table | 1.000 | 1.000 |
| Ethnicity | No | Table | 1.000 | 1.000 |
| Education | No | Table | 1.000 | 1.000 |
| Marital status | No | Table | 1.000 | 1.000 |
| Region | No | Table | 1.000 | 1.000 |
| Income | No | Table | 1.000 | 1.000 |
| Insurance type | No | Table | 1.000 | 1.000 |
| Political affiliation | No | Table | 1.000 | 1.000 |
| Medical history: High cholesterol | No | Table | 1.000 | 1.000 |
| Medical history: Hypertension | No | Table | 1.000 | 1.000 |
| Medical history: Arthritis | No | Table | 1.000 | 1.000 |
| Medical history: Diabetes | No | Table | 1.000 | 1.000 |
| Medical history: Heart failure | No | Table | 1.000 | 1.000 |
| Medical history: Stroke | No | Table | 1.000 | 1.000 |
| Medical history: Bronchitis | No | Table | 1.000 | 1.000 |
| Medical history: Asthma | No | Table | 1.000 | 1.000 |
| Medical history: Depression | No | Table | 1.000 | 1.000 |
| Medical history: Migraine | No | Table | 1.000 | 1.000 |
| Medical history: Cancer | No | Table | 1.000 | 1.000 |
| Medical history: Other | No | Table | --- | 1.000 |
| Medical history: None | No | Table | 1.000 | 1.000 |
| Smoking | No | Table | 1.000 | 1.000 |
| Weight | Yes | Normal | 0.904 | 1.000 |
| Height | Yes | Normal | 0.826 | 1.000 |
| Live alone | No | Table | 1.000 | 1.000 |
| Diagnosed with COVID-19 | No | Table | 1.000 | 1.000 |
| Experienced COVID-19-like symptoms not serious enough to require hospitalization | No | Table | 1.000 | 1.000 |
| Has a family member diagnosed with COVID-19 | No | Table | 1.000 | 1.000 |
| Knows someone with a COVID-19 diagnosis | No | Table | 1.000 | 1.000 |
| Have to defer routine medical care due to COVID-19 |  |  | --- | 1.000 |
| Change of normal diet due to COVID-19 | No | Table | 1.000 | --- |
| Under social distancing | No | Table | 1.000 | --- |
| Under mandatory social distancing | No | Table | --- | 1.000 |
| Practicing social distancing personally | No | Table | --- | 1.000 |
| Supporting social distance policy | No | Table | 1.000 | 1.000 |
| Fear of COVID-19's impact on health (0-10 scale) | No | Table | 1.000 | 1.000 |
| Fear of COVID-19's impact on finances (0-10 scale) | No | Table | 1.000 | 1.000 |
| Average hours of sleep per day | No | Table | 1.000 | 1.000 |
| Change of sleeping pattern | No | Table | 1.000 | 1.000 |
| How many more hours of sleep? | Yes | Gamma | 0.997 | 1.000 |
| How many less hours of sleep? | Yes | Gamma | 0.944 | 1.000 |
| Change of income due to COVID-19 | No | Table | 1.000 | 1.000 |
| Change of spending pattern | No | Table | 1.000 | 1.000 |
| Spend how much more? | Yes | Gamma | 0.996 | 1.000 |
| Spend how much less? | Yes | Gamma | 1.000 | 1.000 |
| Employment status | No | Table | 1.000 | 1.000 |
| Occupation | No | Table | 1.000 | 1.000 |
| Work deemed essential? | No | Table | 1.000 | 1.000 |
| Employment status change due to COVID-19 | No | Table | 1.000 | 1.000 |
| Hours of work missed due to COVID-19 | No | Beta | 0.968 | <0.001 |
| Hours of work missed not due to COVID-19 | No | Beta | 1.000 | <0.001 |
| Average hours of work per day | No | Table | 1.000 | 1.000 |
| COVID-19 impact on working productivity | No | Table | 1.000 | 1.000 |
| COVID-19 impact on non-working productivity | No | Table | 1.000 | 1.000 |
| BRFSS Q1 Did not have enough food or money to get more | No | Table | --- | 1.000 |
| BRFSS Q2 Could not afford to eat balanced meal | No | Table | --- | 1.000 |
| BRFSS Q3 Finances at the end of the month | No | Table | --- | 1.000 |
| BRFSS Q4 Stress within the last 30 days | No | Table | --- | 1.000 |
| CD RISC Q1 Able to bounce back when changes occur | No | Table | --- | 1.000 |
| CD RISC Q2 Tend to bounce back after illness, injury, or other hardships | No | Table | --- | 1.000 |
| Have experienced traumatic events | No | Table | --- | 1.000 |
| PROMIS Q1 Irritated more than people knew in the past seven days | No | Table | --- | 1.000 |
| PROMIS Q2 Felt angry in the past seven days | No | Table | --- | 1.000 |
| PROMIS Q3 Felt like I was ready to explode in the past seven days | No | Table | --- | 1.000 |
| PROMIS Q4 Was grouchy in the past seven days | No | Table | --- | 1.000 |
| PROMIS Q5 Felt annoyed in the past seven days | No | Table | --- | 1.000 |
| PHQ-4 Q1, Feeling nervous, anxious, or on edge | No | Table | 1.000 | 1.000 |
| PHQ-4 Q2, Not being able to stop or control worrying | No | Table | 1.000 | 1.000 |
| PHQ-4 Q3, Feeling down, depressed or hopeless | No | Table | 1.000 | 1.000 |
| PHQ-4 Q4, Little interest or pleasure in doing things | No | Table | 1.000 | 1.000 |
| EQ-5D Mobility | No | Table | 1.000 | 1.000 |
| EQ-5D Self-care | No | Table | 1.000 | 1.000 |
| EQ-5D Usual activities | No | Table | 1.000 | 1.000 |
| EQ-5D Pain/Discomfort | No | Table | 1.000 | 1.000 |
| EQ-5D Anxiety/Depression | No | Table | 1.000 | 1.000 |
| EQ-5D-5L VAS | No | Beta | <0.001 | <0.001 |
| VR12 Q1 Health status | No | Table | 1.000 | 1.000 |
| VR12 Q2a Limit in moderate activities | No | Table | 1.000 | 1.000 |
| VR12 Q2b Limit in climbing several flights of stairs | No | Table | 1.000 | 1.000 |
| VR12 Q3a Accomplished less due to physical health | No | Table | 1.000 | 1.000 |
| VR12 Q3b Limited in work/activities due to physical health | No | Table | 1.000 | 1.000 |
| VR12 Q4a Accomplished less due to emotional problems | No | Table | 1.000 | 1.000 |
| VR12 Q4b Didn’t do work or other activities as carefully due to emotional problems | No | Table | 1.000 | 1.000 |
| VR12 Q5 Pain interfere with normal work | No | Table | 1.000 | 1.000 |
| VR12 Q6a Felt calm and peaceful | No | Table | 1.000 | 1.000 |
| VR12 Q6b Have a lot of energy | No | Table | 1.000 | 1.000 |
| VR12 Q6c Felt downhearted and blue | No | Table | 1.000 | 1.000 |
| VR12 Q7 Physical health or emotional problems interfered with social activities | No | Table | 1.000 | 1.000 |
| VR12 Q8 Physical health compared to one year ago | No | Table | 1.000 | 1.000 |
| VR12 Q9 Emotional problems compared to one year ago | No | Table | 1.000 | 1.000 |
| EQ-5D difficulty | No | Table | 1.000 | 1.000 |
| EQ-5D usefulness | No | Table | 1.000 | 1.000 |
| EQ-5D time used | Yes | Gamma | 1.000 | 1.000 |
| VR12 difficulty | No | Table | 1.000 | 1.000 |
| VR12 usefulness | No | Table | 1.000 | 1.000 |
| VR12 time used | Yes | Gamma | 1.000 | 1.000 |

**Appendix 2. Full sample characteristics in wave 1, by wave 2 participation**

|  | Raw sample | | | | Analytic sample | | |
| --- | --- | --- | --- | --- | --- | --- | --- |
|  | Missing (%) | Not in wave 2 (n=1,267) | In wave 2 (n=1,467) | P-value | Not in wave 2 (n=1,267) | In wave 2 (n=1,467) | P-value |
| Age, years, mean (SD) | 0 (0.0) | 41.6 (14.7) | 43.4 (14.0) | 0.002 | 41.6 (14.7) | 43.4 (14.0) | 0.002 |
| Age group, n (%) | 0 (0.0) |  |  | <0.001 |  |  | <0.001 |
| 18-24 |  | 118 (9.3) | 74 (5.0) |  | 118 (9.3) | 74 (5.0) |  |
| 25-34 |  | 398 (31.4) | 412 (28.1) |  | 398 (31.4) | 412 (28.1) |  |
| 35-44 |  | 287 (22.7) | 373 (25.4) |  | 287 (22.7) | 373 (25.4) |  |
| 45-54 |  | 141 (11.1) | 227 (15.5) |  | 141 (11.1) | 227 (15.5) |  |
| 55-64 |  | 217 (17.1) | 239 (16.3) |  | 217 (17.1) | 239 (16.3) |  |
| ≥65 |  | 106 (8.4) | 142 (9.7) |  | 106 (8.4) | 142 (9.7) |  |
| Gender, n (%) | 9 (0.3) |  |  | 0.835 |  |  | 0.808 |
| Male |  | 611 (48.4) | 724 (49.5) |  | 613 (48.4) | 727 (49.6) |  |
| Female |  | 638 (50.6) | 723 (49.4) |  | 641 (50.6) | 724 (49.4) |  |
| Other |  | 13 (1.0) | 16 (1.1) |  | 13 (1.0) | 16 (1.1) |  |
| Race, n (%) | 0 (0.0) |  |  | 0.129 |  |  | 0.129 |
| White |  | 851 (67.2) | 1,028 (70.1) |  | 851 (67.2) | 1,028 (70.1) |  |
| American Indian or Alaska Native |  | 10 (0.8) | 7 (0.5) |  | 10 (0.8) | 7 (0.5) |  |
| Asian |  | 79 (6.2) | 105 (7.2) |  | 79 (6.2) | 105 (7.2) |  |
| Black or African American |  | 92 (7.3) | 106 (7.2) |  | 92 (7.3) | 106 (7.2) |  |
| Multiple races |  | 213 (16.8) | 195 (13.3) |  | 213 (16.8) | 195 (13.3) |  |
| Native Hawaiian or Other Pacific Islander |  | 3 (0.2) | 1 (0.1) |  | 3 (0.2) | 1 (0.1) |  |
| Other |  | 19 (1.5) | 25 (1.7) |  | 19 (1.5) | 25 (1.7) |  |
| Ethnicity, n (%) | 2 (0.1) |  |  | <0.001 |  |  | <0.001 |
| Non-Hispanic |  | 1,086 (85.7) | 1,358 (92.7) |  | 1,086 (85.7) | 1,360 (92.7) |  |
| Hispanic |  | 170 (13.4) | 98 (6.7) |  | 170 (13.4) | 98 (6.7) |  |
| Prefer not to say |  | 11 (0.9) | 9 (0.6) |  | 11 (0.9) | 9 (0.6) |  |
| Education, n (%) | 0 (0.0) |  |  | 0.130 |  |  | 0.130 |
| Less than high school degree |  | 5 (0.4) | 9 (0.6) |  | 5 (0.4) | 9 (0.6) |  |
| High school degree or equivalent (e.g., GED) |  | 114 (9.0) | 150 (10.2) |  | 114 (9.0) | 150 (10.2) |  |
| Some college but no degree |  | 210 (16.6) | 247 (16.8) |  | 210 (16.6) | 247 (16.8) |  |
| Associate degree |  | 128 (10.1) | 188 (12.8) |  | 128 (10.1) | 188 (12.8) |  |
| Bachelor’s degree |  | 584 (46.1) | 619 (42.2) |  | 584 (46.1) | 619 (42.2) |  |
| Graduate degree |  | 226 (17.8) | 254 (17.3) |  | 226 (17.8) | 254 (17.3) |  |
| Marital status | 0 (0.0) |  |  | 0.348 |  |  | 0.348 |
| Single |  | 483 (38.1) | 587 (40.0) |  | 483 (38.1) | 587 (40.0) |  |
| Married |  | 610 (48.1) | 666 (45.4) |  | 610 (48.1) | 666 (45.4) |  |
| Separated |  | 7 (0.6) | 16 (1.1) |  | 7 (0.6) | 16 (1.1) |  |
| Divorced |  | 117 (9.2) | 148 (10.1) |  | 117 (9.2) | 148 (10.1) |  |
| Widowed |  | 36 (2.8) | 39 (2.7) |  | 36 (2.8) | 39 (2.7) |  |
| Prefer not to say |  | 14 (1.1) | 11 (0.7) |  | 14 (1.1) | 11 (0.7) |  |
| Region | 2 (0.1) |  |  | 0.133 |  |  | 0.132 |
| Northeast |  | 237 (18.7) | 261 (17.8) |  | 237 (18.7) | 261 (17.8) |  |
| Midwest |  | 250 (19.7) | 275 (18.8) |  | 250 (19.7) | 275 (18.7) |  |
| South |  | 436 (34.4) | 568 (38.7) |  | 436 (34.4) | 568 (38.7) |  |
| West |  | 343 (27.1) | 362 (24.7) |  | 344 (27.1) | 363 (24.7) |  |
| Income | 2 (0.1) |  |  | 0.259 |  |  | 0.264 |
| Less than $20,000 |  | 131 (10.3) | 150 (10.2) |  | 131 (10.3) | 150 (10.2) |  |
| $20,000 to $34,999 |  | 201 (15.9) | 222 (15.2) |  | 201 (15.9) | 222 (15.1) |  |
| $35,000 to $49,999 |  | 242 (19.1) | 237 (16.2) |  | 242 (19.1) | 237 (16.2) |  |
| $50,000 to $74,999 |  | 313 (24.7) | 375 (25.6) |  | 313 (24.7) | 375 (25.6) |  |
| $75,000 to $99,999 |  | 184 (14.5) | 256 (17.5) |  | 184 (14.5) | 256 (17.5) |  |
| $100,000 to $149,999 |  | 144 (11.4) | 160 (10.9) |  | 144 (11.4) | 162 (11.0) |  |
| Over $150,000 |  | 52 (4.1) | 65 (4.4) |  | 52 (4.1) | 65 (4.4) |  |
| Insurance | 1,945 (71.1) |  |  | <0.001 |  |  | 0.137 |
| Commercial or private |  | 162 (40.5) | 213 (54.8) |  | 578 (45.6) | 726 (49.5) |  |
| Medicare |  | 76 (19.0) | 38 (9.8) |  | 192 (15.2) | 186 (12.7) |  |
| Medicaid/ACA |  | 74 (18.5) | 68 (17.5) |  | 244 (19.3) | 269 (18.3) |  |
| Other |  | 88 (22.0) | 70 (18.0) |  | 253 (19.3) | 286 (19.5) |  |
| Political affiliation | 69 (2.5) |  |  | 0.854 |  |  | 0.858 |
| Republican |  | 364 (29.4) | 409 (28.6) |  | 373 (29.4) | 421 (28.7) |  |
| Democrat |  | 580 (46.9) | 665 (46.5) |  | 596 (47.0) | 681 (46.4) |  |
| Independent |  | 282 (22.8) | 345 (24.1) |  | 288 (22.7) | 354 (24.1) |  |
| None of the above |  | 10 (0.8) | 10 (0.7) |  | 10 (0.8) | 11 (0.7) |  |
| Medical history: High cholesterol | 198 (7.2) | 190 (15.9) | 216 (16.1) | 0.887 | 199 (15.7) | 234 (16.0) | 0.861 |
| Medical history: Hypertension | 198 (7.2) | 215 (18.0) | 222 (16.6) | 0.339 | 224 (17.7) | 242 (16.5) | 0.412 |
| Medical history: Arthritis | 198 (7.2) | 126 (10.5) | 150 (11.2) | 0.604 | 132 (10.4) | 165 (11.2) | 0.487 |
| Medical history: Diabetes | 198 (7.2) | 124 (10.4) | 90 (6.7) | <0.001 | 129 (10.2) | 98 (6.7) | <0.001 |
| Medical history: Heart failure | 198 (7.2) | 30 (2.5) | 26 (1.9) | 0.328 | 31 (2.4) | 28 (1.9) | 0.334 |
| Medical history: Stroke | 198 (7.2) | 41 (3.4) | 20 (1.5) | 0.001 | 42 (3.3) | 22 (1.5) | 0.002 |
| Medical history: Bronchitis | 198 (7.2) | 77 (6.4) | 78 (5.8) | 0.511 | 80 (6.3) | 83 (5.7) | 0.470 |
| Medical history: Asthma | 198 (7.2) | 174 (14.6) | 152 (11.3) | 0.015 | 181 (14.3) | 170 (11.6) | 0.036 |
| Medical history: Depression | 198 (7.2) | 284 (23.8) | 312 (23.3) | 0.767 | 300 (23.7) | 341 (23.2) | 0.790 |
| Medical history: Migraine | 198 (7.2) | 112 (9.4) | 136 (10.1) | 0.515 | 117 (9.2) | 147 (10.0) | 0.488 |
| Medical history: Cancer | 198 (7.2) | 144 (12.1) | 126 (9.4) | 0.031 | 150 (11.8) | 141 (9.6) | 0.060 |
| Medical history: None | 545 (19.9) | 390 (38.7) | 492 (41.7) | 0.158 | 495 (39.1) | 612 (41.7) | 0.159 |
| Smoking history | 545 (19.9) |  |  | 0.009 |  |  | 0.096 |
| Currently |  | 191 (18.9) | 174 (14.7) |  | 226 (17.8) | 226 (15.4) |  |
| In the past |  | 321 (31.8) | 360 (30.5) |  | 404 (31.9) | 448 (30.5) |  |
| Never |  | 496 (49.2) | 647 (54.8) |  | 637 (50.3) | 793 (54.1) |  |
| BMI category | 545 (19.9) |  |  | <0.001 |  |  | 0.010 |
| Underweight |  | 104 (10.3) | 58 (4.9) |  | 135 (10.7) | 105 (7.2) |  |
| Normal weight |  | 379 (37.6) | 495 (41.9) |  | 477 (37.6) | 586 (39.9) |  |
| Overweight |  | 286 (28.4) | 364 (30.8) |  | 374 (29.5) | 462 (31.5) |  |
| Obesity |  | 239 (23.7) | 264 (22.4) |  | 281 (22.2) | 314 (21.4) |  |
| Live alone | 461 (16.9) | 295 (27.6) | 295 (24.5) | 0.098 | 343 (27.1) | 371 (25.3) | 0.290 |
| Diagnosed with COVID-19 | 0 (0.0) | 63 (5.0) | 29 (2.0) | <0.001 | 63 (5.0) | 29 (2.0) | <0.001 |
| Experienced COVID-19-like symptoms not serious enough to require hospitalization | 92 (3.4) | 144 (12.0) | 108 (7.5) | <0.001 | 148 (11.7) | 111 (7.6) | <0.001 |
| Has a family member diagnosed with COVID-19 | 2 (0.1) | 101 (8.0) | 86 (5.9) | 0.029 | 102 (8.1) | 86 (5.9) | 0.024 |
| Knows someone with a COVID-19 diagnosis | 1 (0.0) | 305 (24.1) | 265 (18.1) | <0.001 | 305 (24.1) | 266 (18.1) | <0.001 |
| Change of normal diet due to COVID-19 | 0 (0.0) | 503 (39.7) | 429 (29.2) | <0.001 | 503 (39.7) | 429 (29.2) | <0.001 |
| Under social distancing | 461 (16.9) | 975 (91.1) | 1,087 (90.4) | 0.531 | 1,156 (91.2) | 1,322 (90.0) | 0.315 |
| Fear of COVID-19's impact on health (0-10 scale) | 0 (0.0) | 5.3 (2.9) | 5.1 (3.0) | 0.029 | 5.3 (2.9) | 5.1 (3.0) | 0.029 |
| Fear of COVID-19's impact on finances (0-10 scale) | 0 (0.0) | 5.9 (3.0) | 5.7 (3.0) | 0.054 | 5.9 (3.0) | 5.7 (3.0) | 0.054 |
| Supporting social distance policy (0-10 scale) | 461 (16.9) | 8.2 (2.5) | 8.5 (2.5) | 0.006 | 8.3 (2.5) | 8.5 (2.5) | 0.026 |
| Average hours of sleep per day (1-15 scale) | 0 (0.0) | 7.4 (1.7) | 7.1 (1.4) | <0.001 | 7.4 (1.7) | 7.1 (1.4) | <0.001 |
| Change of sleeping pattern | 0 (0.0) |  |  | <0.001 |  |  | <0.001 |
| More |  | 336 (26.5) | 274 (18.7) |  | 336 (26.5) | 274 (18.7) |  |
| Less |  | 247 (19.5) | 284 (19.4) |  | 247 (19.5) | 284 (19.4) |  |
| No change |  | 684 (54.0) | 909 (62.0) |  | 684 (54.0) | 909 (62.0) |  |
| Change of income due to COVID-19 | 545 (19.9) |  |  | <0.001 |  |  | <0.001 |
| Increased |  | 45 (4.5) | 45 (3.8) |  | 577 (45.5) | 803 (54.7) |  |
| Reduced |  | 515 (51.1) | 476 (40.3) |  | 636 (50.2) | 604 (41.2) |  |
| No change |  | 448 (44.4) | 660 (55.9) |  | 54 (4.3) | 60 (4.1) |  |
| Change of spending pattern | 0 (0.0) |  |  | 0.157 |  |  | 0.157 |
| More |  | 232 (18.3) | 241 (16.4) |  | 232 (18.3) | 241 (16.4) |  |
| Less |  | 674 (53.2) | 763 (52.0) |  | 674 (53.2) | 763 (52.0) |  |
| No change |  | 361 (28.5) | 463 (31.6) |  | 361 (28.5) | 463 (31.6) |  |
| Employment status | 8 (0.3) |  |  | 0.221 |  |  | 0.190 |
| Employed full-time |  | 713 (56.5) | 883 (60.4) |  | 715 (56.4) | 886 (60.4) |  |
| Employed part-time |  | 206 (16.3) | 231 (15.8) |  | 206 (16.3) | 232 (15.8) |  |
| Unemployed, seeking employment |  | 91 (7.2) | 84 (5.7) |  | 91 (7.2) | 84 (5.7) |  |
| Unemployed, not seeking employment |  | 53 (4.2) | 52 (3.6) |  | 53 (4.2) | 52 (3.5) |  |
| Student |  | 38 (3.0) | 26 (1.8) |  | 39 (3.1) | 26 (1.8) |  |
| Retired |  | 105 (8.3) | 120 (8.2) |  | 106 (8.4) | 120 (8.2) |  |
| On disability and can't work |  | 15 (1.2) | 19 (1.3) |  | 15 (1.2) | 19 (1.3) |  |
| Homemaker or stay at home parent |  | 39 (3.1) | 47 (3.2) |  | 39 (3.1) | 47 (3.2) |  |
| Don't know |  | 3 (0.2) | 1 (0.1) |  | 3 (0.2) | 1 (0.1) |  |
| Occupation | 606 (22.2) |  |  | 0.138 |  |  | 0.244 |
| Management |  | 103 (10.6) | 101 (8.7) |  | 131 (10.3) | 139 (9.5) |  |
| Business and Financial Operations |  | 110 (11.3) | 143 (12.3) |  | 132 (10.4) | 180 (12.3) |  |
| Computer and Mathematical |  | 118 (12.2) | 124 (10.7) |  | 154 (12.2) | 168 (11.5) |  |
| Architecture and Engineering |  | 22 (2.3) | 17 (1.5) |  | 25 (2.0) | 23 (1.6) |  |
| Life, Physical, and Social Science |  | 11 (1.1) | 34 (2.9) |  | 16 (1.3) | 39 (2.7) |  |
| Community and Social Service |  | 16 (1.6) | 17 (1.5) |  | 23 (1.8) | 22 (1.5) |  |
| Legal |  | 18 (1.9) | 16 (1.4) |  | 21 (1.7) | 18 (1.2) |  |
| Educational Instruction and Library |  | 73 (7.5) | 102 (8.8) |  | 103 (8.1) | 119 (8.1) |  |
| Arts, Design, Entertainment, Sports, and Media |  | 42 (4.3) | 79 (6.8) |  | 57 (4.5) | 88 (6.0) |  |
| Healthcare Practitioners and Technicians |  | 35 (3.6) | 37 (3.2) |  | 48 (3.8) | 48 (3.3) |  |
| Healthcare Support |  | 24 (2.5) | 36 (3.1) |  | 36 (2.8) | 46 (3.1) |  |
| Protective Service |  | 4 (0.4) | 7 (0.6) |  | 4 (0.3) | 8 (0.5) |  |
| Food Preparation and Serving |  | 28 (2.9) | 32 (2.8) |  | 33 (2.6) | 39 (2.7) |  |
| Building and Grounds Cleaning and Maintenance |  | 7 (0.7) | 8 (0.7) |  | 9 (0.7) | 11 (0.7) |  |
| Personal Care and Service |  | 18 (1.9) | 18 (1.6) |  | 24 (1.9) | 26 (1.8) |  |
| Sales |  | 77 (7.9) | 101 (8.7) |  | 104 (8.2) | 128 (8.7) |  |
| Office and Administrative Support |  | 106 (10.9) | 115 (9.9) |  | 144 (11.4) | 145 (9.9) |  |
| Farming, Fishing, and Forestry |  | 8 (0.8) | 8 (0.7) |  | 9 (0.7) | 11 (0.7) |  |
| Construction and Extraction |  | 22 (2.3) | 14 (1.2) |  | 27 (2.1) | 17 (1.2) |  |
| Installation, Maintenance, and Repair |  | 14 (1.4) | 12 (1.0) |  | 16 (1.3) | 16 (1.1) |  |
| Production and Manufacturing |  | 32 (3.3) | 33 (2.8) |  | 46 (3.6) | 45 (3.1) |  |
| Transportation and Material Moving |  | 20 (2.1) | 32 (2.8) |  | 27 (2.1) | 38 (2.6) |  |
| Military |  | 6 (0.6) | 4 (0.3) |  | 9 (0.7) | 5 (0.3) |  |
| Research |  | 19 (2.0) | 30 (2.6) |  | 20 (1.6) | 38 (2.6) |  |
| Don't know |  | 37 (3.8) | 38 (3.3) |  | 49 (3.9) | 50 (3.4) |  |
| Employment status change due to COVID-19 | 3 (0.1) |  |  | 0.003 |  |  |  |
| No change |  | 634 (50.0) | 798 (54.5) |  | 634 (50.0) | 799 (54.5) |  |
| Lost job |  | 93 (7.3) | 67 (4.6) |  | 93 (7.3) | 67 (4.6) |  |
| Work from home |  | 405 (32.0) | 470 (32.1) |  | 405 (32.0) | 471 (32.1) |  |
| Laid off temporarily |  | 135 (10.7) | 129 (8.8) |  | 135 (10.7) | 130 (8.9) |  |
| Work deemed essential? | 126 (4.6) |  |  | 0.016 |  |  | 0.005 |
| Yes |  | 437 (36.0) | 439 (31.5) |  | 459 (36.2) | 457 (31.2) |  |
| No |  | 742 (61.2) | 927 (66.5) |  | 773 (61.0) | 981 (66.9) |  |
| Don't know |  | 34 (2.8) | 29 (2.1) |  | 35 (2.8) | 29 (2.0) |  |
| Hours of work missed due to COVID-19 | 3 (0.1) | 6.1 (12.6) | 4.2 (11.0) | <0.001 | 6.1 (12.6) | 4.2 (10.9) | <0.001 |
| Hours of work missed not due to COVID-19 | 3 (0.1) | 2.0 (6.6) | 1.3 (6.0) | 0.006 | 2.0 (6.6) | 1.3 (6.0) | 0.006 |
| Average hours of work per day | 2 (0.1) | 4.6 (3.7) | 5.0 (3.7) | 0.008 | 4.6 (3.7) | 5.0 (3.7) | 0.007 |
| COVID-19 impact on productivity | 1 (0.0) | 4.1 (2.8) | 3.4 (2.6) | <0.001 | 4.1 (2.8) | 3.4 (2.6) | <0.001 |
| PHQ-4 score | 5 (0.2) |  |  | <0.001 |  |  | <0.001 |
| 0-2 |  | 521 (41.2) | 726 (49.6) |  | 521 (41.4) | 726 (49.5) |  |
| 3-5 |  | 292 (23.1) | 344 (23.5) |  | 294 (23.2) | 346 (23.6) |  |
| 6-8 |  | 292 (23.1) | 243 (16.6) |  | 292 (23.0) | 244 (16.6) |  |
| 9-12 |  | 160 (12.6) | 151 (10.3) |  | 160 (12.6) | 151 (10.3) |  |
| EQ-5D-5L Mobility | 0 (0.0) |  |  | <0.001 |  |  | <0.001 |
| No problems walking |  | 951 (75.1) | 1,214 (82.8) |  | 951 (75.1) | 1,214 (82.8) |  |
| Slight problems walking |  | 175 (13.8) | 144 (9.8) |  | 175 (13.8) | 144 (9.8) |  |
| Moderate problems walking |  | 94 (7.4) | 84 (5.7) |  | 94 (7.4) | 84 (5.7) |  |
| Severe problems walking |  | 37 (2.9) | 16 (1.1) |  | 37 (2.9) | 16 (1.1) |  |
| Unable to walk |  | 10 (0.8) | 9 (0.6) |  | 10 (0.8) | 9 (0.6) |  |
| EQ-5D-5L Self-care | 0 (0.0) |  |  | <0.001 |  |  | <0.001 |
| No problems washing or dressing myself |  | 1,059 (83.6) | 1,316 (89.7) |  | 1,059 (83.6) | 1,316 (89.7) |  |
| Slight problems washing or dressing myself |  | 107 (8.4) | 83 (5.7) |  | 107 (8.4) | 83 (5.7) |  |
| Moderate problems washing or dressing myself |  | 70 (5.5) | 47 (3.2) |  | 70 (5.5) | 47 (3.2) |  |
| Severe problems washing or dressing myself |  | 27 (2.1) | 14 (1.0) |  | 27 (2.1) | 14 (1.0) |  |
| Unable to wash or dress myself |  | 4 (0.3) | 7 (0.5) |  | 4 (0.3) | 7 (0.5) |  |
| EQ-5D-5L Usual activities | 0 (0.0) |  |  | <0.001 |  |  | <0.001 |
| No problems doing usual activities |  | 881 (69.5) | 1,138 (77.6) |  | 881 (69.5) | 1,138 (77.6) |  |
| Slight problems doing usual activities |  | 228 (18.0) | 195 (13.3) |  | 228 (18.0) | 195 (13.3) |  |
| Moderate problems doing usual activities |  | 108 (8.5) | 104 (7.1) |  | 108 (8.5) | 104 (7.1) |  |
| Severe problems doing usual activities |  | 40 (3.2) | 24 (1.6) |  | 40 (3.2) | 24 (1.6) |  |
| Unable to do usual activities |  | 10 (0.8) | 6 (0.4) |  | 10 (0.8) | 6 (0.4) |  |
| EQ-5D-5L Pain/Discomfort | 2 (0.1) |  |  | 0.121 |  |  | 0.121 |
| No pain or discomfort |  | 642 (50.7) | 805 (54.9) |  | 642 (50.7) | 805 (54.9) |  |
| Slight pain or discomfort |  | 405 (32.0) | 411 (28.0) |  | 405 (32.0) | 411 (28.0) |  |
| Moderate pain or discomfort |  | 169 (13.3) | 204 (13.9) |  | 170 (13.4) | 205 (14.0) |  |
| Severe pain or discomfort |  | 45 (3.6) | 42 (2.9) |  | 45 (3.6) | 42 (2.9) |  |
| Extreme pain or discomfort |  | 5 (0.4) | 4 (0.3) |  | 5 (0.4) | 4 (0.3) |  |
| EQ-5D-5L Anxiety/Depression | 0 (0.0) |  |  | 0.028 |  |  | 0.028 |
| Not anxious or depressed |  | 465 (36.7) | 615 (41.9) |  | 465 (36.7) | 615 (41.9) |  |
| Slightly anxious or depressed |  | 407 (32.1) | 443 (30.2) |  | 407 (32.1) | 443 (30.2) |  |
| Moderately anxious or depressed |  | 303 (23.9) | 293 (20.0) |  | 303 (23.9) | 293 (20.0) |  |
| Severely anxious or depressed |  | 47 (3.7) | 62 (4.2) |  | 47 (3.7) | 62 (4.2) |  |
| Extremely anxious or depressed |  | 45 (3.6) | 54 (3.7) |  | 45 (3.6) | 54 (3.7) |  |
| EQ-5D-5L VAS | 4 (0.1) | 73.9 (19.2) | 75.2 (19.3) | 0.077 | 73.9 (19.2) | 75.1 (19.3) | 0.088 |
| EQ-5D-5L utility | 2 (0.1) | 0.8 (0.2) | 0.8 (0.2) | <0.001 | 0.8 (0.2) | 0.8 (0.2) | <0.001 |
| VR12 Q1 General Health status | 1 (0.0) |  |  | 0.991 |  |  | 0.993 |
| Excellent |  | 162 (12.8) | 195 (13.3) |  | 162 (12.8) | 195 (13.3) |  |
| Very good |  | 474 (37.4) | 545 (37.2) |  | 474 (37.4) | 545 (37.2) |  |
| Good |  | 432 (34.1) | 492 (33.5) |  | 432 (34.1) | 492 (33.5) |  |
| Fair |  | 174 (13.7) | 207 (14.1) |  | 175 (13.8) | 207 (14.1) |  |
| Poor |  | 24 (1.9) | 28 (1.9) |  | 24 (1.9) | 28 (1.9) |  |
| VR12 Q2a Limit in moderate activities | 1 (0.0) |  |  | <0.001 |  |  | <0.001 |
| Limited a lot |  | 71 (5.6) | 74 (5.0) |  | 71 (5.6) | 74 (5.0) |  |
| Limited a little |  | 322 (25.4) | 236 (16.1) |  | 322 (25.4) | 236 (16.1) |  |
| Not limited at all |  | 874 (69.0) | 1,156 (78.9) |  | 874 (69.0) | 1,157 (78.9) |  |
| VR12 Q2b Limit in climbing several flights of stairs | 8 (0.3) |  |  | <0.001 |  |  | <0.001 |
| Limited a lot |  | 154 (12.2) | 120 (8.2) |  | 154 (12.2) | 120 (8.2) |  |
| Limited a little |  | 302 (23.9) | 290 (19.8) |  | 303 (23.9) | 291 (19.8) |  |
| Not limited at all |  | 806 (63.9) | 1,054 (72.0) |  | 810 (63.9) | 1,056 (72.0) |  |
| VR12 Q3a Accomplished less due to physical health | 5 (0.2) |  |  | <0.001 |  |  | <0.001 |
| None of the time |  | 661 (52.2) | 943 (64.5) |  | 661 (52.2) | 945 (64.4) |  |
| A little of the time |  | 232 (18.3) | 216 (14.8) |  | 232 (18.3) | 217 (14.8) |  |
| Some of the time |  | 228 (18.0) | 190 (13.0) |  | 228 (18.0) | 191 (13.0) |  |
| Most of the time |  | 110 (8.7) | 83 (5.7) |  | 111 (8.8) | 83 (5.7) |  |
| All of the time |  | 35 (2.8) | 31 (2.1) |  | 35 (2.8) | 31 (2.1) |  |
| VR12 Q3b Limited in work/activities due to physical health | 4 (0.1) |  |  | <0.001 |  |  | <0.001 |
| None of the time |  | 688 (54.3) | 976 (66.7) |  | 688 (54.3) | 978 (66.7) |  |
| A little of the time |  | 212 (16.7) | 220 (15.0) |  | 212 (16.7) | 220 (15.0) |  |
| Some of the time |  | 195 (15.4) | 151 (10.3) |  | 195 (15.4) | 152 (10.4) |  |
| Most of the time |  | 127 (10.0) | 75 (5.1) |  | 128 (10.1) | 75 (5.1) |  |
| All of the time |  | 44 (3.5) | 42 (2.9) |  | 44 (3.5) | 42 (2.9) |  |
| VR12 Q4a Accomplished less due to emotional problems | 4 (0.1) |  |  | <0.001 |  |  | <0.001 |
| None of the time |  | 485 (38.3) | 730 (49.8) |  | 485 (38.3) | 731 (49.8) |  |
| A little of the time |  | 312 (24.7) | 322 (22.0) |  | 312 (24.6) | 323 (22.0) |  |
| Some of the time |  | 280 (22.1) | 256 (17.5) |  | 281 (22.1) | 256 (17.5) |  |
| Most of the time |  | 140 (11.1) | 124 (8.5) |  | 141 (11.1) | 124 (8.5) |  |
| All of the time |  | 48 (3.8) | 33 (2.3) |  | 48 (3.8) | 33 (2.2) |  |
| VR12 Q4b Didn’t do work or other activities as carefully due to emotional problems | 4 (0.1) |  |  | <0.001 |  |  | <0.001 |
| None of the time |  | 589 (46.6) | 842 (57.5) |  | 589 (46.5) | 844 (57.5) |  |
| A little of the time |  | 298 (23.6) | 301 (20.5) |  | 298 (23.5) | 301 (20.5) |  |
| Some of the time |  | 242 (19.1) | 222 (15.2) |  | 243 (19.2) | 222 (15.1) |  |
| Most of the time |  | 102 (8.1) | 78 (5.3) |  | 103 (8.1) | 78 (5.3) |  |
| All of the time |  | 34 (2.7) | 22 (1.5) |  | 34 (2.7) | 22 (1.5) |  |
| VR12 Q5 Pain interfere with normal work | 0 (0.0) |  |  | <0.001 |  |  | <0.001 |
| Not at all |  | 650 (51.3) | 869 (59.2) |  | 650 (51.3) | 869 (59.2) |  |
| A little bit |  | 358 (28.3) | 382 (26.0) |  | 358 (28.3) | 382 (26.0) |  |
| Moderately |  | 178 (14.0) | 159 (10.8) |  | 178 (14.0) | 159 (10.8) |  |
| Quite a bit |  | 65 (5.1) | 51 (3.5) |  | 65 (5.1) | 51 (3.5) |  |
| Extremely |  | 16 (1.3) | 6 (0.4) |  | 16 (1.3) | 6 (0.4) |  |
| VR12 Q6a Felt calm and peaceful | 1 (0.0) |  |  | 0.033 |  |  | 0.032 |
| All of the time |  | 74 (5.8) | 116 (7.9) |  | 74 (5.8) | 116 (7.9) |  |
| Most of the time |  | 345 (27.3) | 447 (30.5) |  | 345 (27.2) | 447 (30.5) |  |
| A good bit of the time |  | 239 (18.9) | 278 (19.0) |  | 239 (18.9) | 278 (19.0) |  |
| Some of the time |  | 286 (22.6) | 275 (18.7) |  | 286 (22.6) | 275 (18.7) |  |
| A little of the time |  | 256 (20.2) | 277 (18.9) |  | 257 (20.3) | 277 (18.9) |  |
| None of the time |  | 66 (5.2) | 74 (5.0) |  | 66 (5.2) | 74 (5.0) |  |
| VR12 Q6b Have a lot of energy | 5 (0.2) |  |  | <0.001 |  |  | <0.001 |
| All of the time |  | 56 (4.4) | 70 (4.8) |  | 56 (4.4) | 70 (4.8) |  |
| Most of the time |  | 214 (16.9) | 292 (19.9) |  | 214 (16.9) | 292 (19.9) |  |
| A good bit of the time |  | 261 (20.6) | 354 (24.2) |  | 261 (20.6) | 355 (24.2) |  |
| Some of the time |  | 370 (29.3) | 326 (22.3) |  | 371 (29.3) | 327 (22.3) |  |
| A little of the time |  | 270 (21.4) | 285 (19.5) |  | 271 (21.4) | 285 (19.4) |  |
| None of the time |  | 93 (7.4) | 138 (9.4) |  | 94 (7.4) | 138 (9.4) |  |
| VR12 Q6c Felt downhearted and blue | 4 (0.1) |  |  | <0.001 |  |  | 0.001 |
| All of the time |  | 48 (3.8) | 69 (4.7) |  | 48 (3.8) | 69 (4.7) |  |
| Most of the time |  | 115 (9.1) | 118 (8.0) |  | 115 (9.1) | 118 (8.0) |  |
| A good bit of the time |  | 174 (13.8) | 168 (11.5) |  | 174 (13.7) | 169 (11.5) |  |
| Some of the time |  | 262 (20.7) | 269 (18.3) |  | 262 (20.7) | 269 (18.3) |  |
| A little of the time |  | 401 (31.7) | 436 (29.7) |  | 402 (31.7) | 436 (29.7) |  |
| None of the time |  | 264 (20.9) | 406 (27.7) |  | 266 (21.0) | 406 (27.7) |  |
| VR12 Q7 Physical health or emotional problems interfered with social activities | 2 (0.1) |  |  | <0.001 |  |  | <0.001 |
| All of the time |  | 65 (5.1) | 47 (3.2) |  | 65 (5.1) | 47 (3.2) |  |
| Most of the time |  | 115 (9.1) | 103 (7.0) |  | 115 (9.1) | 103 (7.0) |  |
| Some of the time |  | 258 (20.4) | 243 (16.6) |  | 258 (20.4) | 243 (16.6) |  |
| A little of the time |  | 264 (20.9) | 296 (20.2) |  | 264 (20.8) | 296 (20.2) |  |
| None of the time |  | 564 (44.5) | 777 (53.0) |  | 565 (44.6) | 778 (53.0) |  |
| VR12 Q8 Physical health compared to one year ago | 2 (0.1) |  |  | 0.005 |  |  | 0.005 |
| Much better |  | 97 (7.7) | 70 (4.8) |  | 97 (7.7) | 70 (4.8) |  |
| Slightly better |  | 221 (17.4) | 236 (16.1) |  | 221 (17.4) | 236 (16.1) |  |
| About the same |  | 741 (58.5) | 938 (64.0) |  | 741 (58.5) | 938 (63.9) |  |
| Slightly worse |  | 178 (14.0) | 195 (13.3) |  | 178 (14.0) | 197 (13.4) |  |
| Much worse |  | 30 (2.4) | 26 (1.8) |  | 30 (2.4) | 26 (1.8) |  |
| VR12 Q9 Emotional problems compared to one year ago | 0 (0.0) |  |  | <0.001 |  |  | <0.001 |
| Much better |  | 125 (9.9) | 79 (5.4) |  | 125 (9.9) | 79 (5.4) |  |
| Slightly better |  | 216 (17.0) | 199 (13.6) |  | 216 (17.0) | 199 (13.6) |  |
| About the same |  | 541 (42.7) | 717 (48.9) |  | 541 (42.7) | 717 (48.9) |  |
| Slightly worse |  | 307 (24.2) | 379 (25.8) |  | 307 (24.2) | 379 (25.8) |  |
| Much worse |  | 78 (6.2) | 93 (6.3) |  | 78 (6.2) | 93 (6.3) |  |
| Self-rated survey difficulty (0-10 scale)  Self-rated survey usefulness (0-10 scale) | 0 (0.0)  0 (0.0) | 1.6 (2.2)  5.9 (2.4) | 1.0 (1.8)  6.0 (2.5) | <0.001  0.734 | 1.6 (2.2)  5.9 (2.4) | 1.0 (1.8)  6.0 (2.5) | <0.001  0.749 |
| Survey time used | 0 (0.0) | 3.9 (38.7) | 2.8 (2.2) | 0.282 | 2.3 (1.2) | 2.3 (1.1) | 0.757 |

Note: Due to outlier removal, differences may exist before and after processing even if there was no missing in the variable.

**Appendix 3. Full sample characteristics in wave 2, by wave 3 participation**

|  | Raw sample | | | | Analytic sample | | |
| --- | --- | --- | --- | --- | --- | --- | --- |
|  | Missing (%) | Not in wave 3 (n=1,490) | In wave 3 (n=964) | P-value | Not in wave 3 (n=1,490) | In wave 3 (n=964) | P-value |
| Age, years, mean (SD) | 0 (0.0) | 37.7 (11.9) | 45.3 (14.0) | <0.001 | 37.7 (11.9) | 45.3 (14.0) | <0.001 |
| Age group, n (%) | 0 (0.0) |  |  | <0.001 |  |  | <0.001 |
| 18-24 |  | 71 (4.8) | 30 (3.1) |  | 71 (4.8) | 30 (3.1) |  |
| 25-34 |  | 679 (45.6) | 232 (24.1) |  | 679 (45.6) | 232 (24.1) |  |
| 35-44 |  | 346 (23.2) | 250 (25.9) |  | 346 (23.2) | 250 (25.9) |  |
| 45-54 |  | 216 (14.5) | 164 (17.0) |  | 216 (14.5) | 164 (17.0) |  |
| 55-64 |  | 129 (8.7) | 170 (17.6) |  | 129 (8.7) | 170 (17.6) |  |
| ≥65 |  | 49 (3.3) | 118 (12.2) |  | 49 (3.3) | 118 (12.2) |  |
| Gender, n (%) | 1,223 (49.8) |  |  | 0.060 |  |  | 0.343 |
| Male |  | 624 (57.7) | 72 (48.3) |  | 850 (57.0) | 521 (54.0) |  |
| Female |  | 453 (41.9) | 77 (51.7) |  | 634 (42.6) | 439 (45.5) |  |
| Other |  | 5 (0.5) | - |  | 6 (0.4) | 4 (0.4) |  |
| Race, n (%) | 0 (0.0) |  |  | <0.001 |  |  | <0.001 |
| White |  | 1,126 (75.6) | 752 (78.0) |  | 1,126 (75.6) | 752 (78.0) |  |
| American Indian or Alaska Native |  | 19 (1.3) | 5 (0.5) |  | 19 (1.3) | 5 (0.5) |  |
| Asian |  | 59 (4.0) | 86 (8.9) |  | 59 (4.0) | 86 (8.9) |  |
| Black or African American |  | 235 (15.8) | 72 (7.5) |  | 235 (15.8) | 72 (7.5) |  |
| Multiple races |  | 35 (2.3) | 30 (3.1) |  | 35 (2.3) | 30 (3.1) |  |
| Native Hawaiian or Other Pacific Islander |  | - | 1 (0.1) |  | - | 1 (0.1) |  |
| Other |  | 16 (1.1) | 18 (1.9) |  | 16 (1.1) | 18 (1.9) |  |
| Ethnicity, n (%) | 0 (0.0) |  |  | <0.001 |  |  | <0.001 |
| Non-Hispanic |  | 1,071 (71.9) | 916 (95.0) |  | 1,071 (71.9) | 916 (95.0) |  |
| Hispanic |  | 397 (26.6) | 43 (4.5) |  | 397 (26.6) | 43 (4.5) |  |
| Prefer not to say |  | 22 (1.5) | 5 (0.5) |  | 22 (1.5) | 5 (0.5) |  |
| Education, n (%) | 0 (0.0) |  |  | <0.001 |  |  | <0.001 |
| Less than high school degree |  | 3 (0.2) | 4 (0.4) |  | 3 (0.2) | 4 (0.4) |  |
| High school degree or equivalent (e.g., GED) |  | 78 (5.2) | 100 (10.4) |  | 78 (5.2) | 100 (10.4) |  |
| Some college but no degree |  | 125 (8.4) | 150 (15.6) |  | 125 (8.4) | 150 (15.6) |  |
| Associate degree |  | 113 (7.6) | 124 (12.9) |  | 113 (7.6) | 124 (12.9) |  |
| Bachelor’s degree |  | 793 (53.2) | 410 (42.5) |  | 793 (53.2) | 410 (42.5) |  |
| Graduate degree  Don’t know |  | 377 (25.3)  1 (0.1) | 176 (18.3)  - |  | 377 (25.3)  1 (0.1) | 176 (18.3)  - |  |
| Marital status | 0 (0.0) |  |  | <0.001 |  |  | <0.001 |
| Single |  | 403 (27.0) | 382 (39.6) |  | 403 (27.0) | 382 (39.6) |  |
| Married |  | 1,013 (68.0) | 411 (42.6) |  | 1,013 (68.0) | 411 (42.6) |  |
| Separated |  | 16 (1.1) | 13 (1.3) |  | 16 (1.1) | 13 (1.3) |  |
| Divorced |  | 48 (3.2) | 122 (12.7) |  | 48 (3.2) | 122 (12.7) |  |
| Widowed |  | 7 (0.5) | 28 (2.9) |  | 7 (0.5) | 28 (2.9) |  |
| Prefer not to say |  | 3 (0.2) | 8 (0.8) |  | 3 (0.2) | 8 (0.8) |  |
| Region | 2 (0.1) |  |  | <0.001 |  |  | <0.001 |
| Northeast |  | 273 (18.5) | 165 (17.1) |  | 275 (18.5) | 165 (17.1) |  |
| Midwest |  | 230 (15.5) | 195 (20.2) |  | 230 (15.4) | 195 (20.2) |  |
| South |  | 529 (35.6) | 370 (38.4) |  | 529 (35.5) | 370 (38.4) |  |
| West |  | 454 (30.5) | 234 (24.3) |  | 456 (30.6) | 234 (24.3) |  |
| Income | 1 (0.0) |  |  | <0.001 |  |  | <0.001 |
| Less than $20,000 |  | 109 (7.3) | 100 (10.4) |  | 109 (7.3) | 100 (10.4) |  |
| $20,000 to $34,999 |  | 196 (13.2) | 155 (16.1) |  | 196 (13.2) | 155 (16.1) |  |
| $35,000 to $49,999 |  | 334 (22.4) | 148 (15.4) |  | 334 (22.4) | 148 (15.4) |  |
| $50,000 to $74,999 |  | 465 (31.2) | 237 (24.6) |  | 465 (31.2) | 237 (24.6) |  |
| $75,000 to $99,999 |  | 260 (17.4) | 169 (17.5) |  | 260 (17.4) | 169 (17.5) |  |
| $100,000 to $149,999 |  | 86 (5.8) | 113 (11.7) |  | 86 (5.8) | 114 (11.8) |  |
| Over $150,000 |  | 40 (2.7) | 41 (4.3) |  | 40 (2.7) | 41 (4.3) |  |
| Insurance | 0 (0.0) |  |  | <0.001 |  |  | <0.001 |
| Commercial or private |  | 419 (28.1) | 494 (51.2) |  | 419 (28.1) | 494 (51.2) |  |
| Medicare |  | 593 (39.8) | 154 (16.0) |  | 593 (39.8) | 154 (16.0) |  |
| Medicaid/ACA |  | 256 (17.2) | 194 (20.1) |  | 256 (17.2) | 194 (20.1) |  |
| Other |  | 222 (14.9) | 122 (12.7) |  | 222 (14.9) | 122 (12.7) |  |
| Political affiliation | 0 (0.0) |  |  | <0.001 |  |  | <0.001 |
| Republican |  | 589 (39.5) | 248 (25.7) |  | 589 (39.5) | 248 (25.7) |  |
| Democrat |  | 596 (40.0) | 451 (46.8) |  | 596 (40.0) | 451 (46.8) |  |
| Independent |  | 278 (18.7) | 225 (23.3) |  | 278 (18.7) | 225 (23.3) |  |
| None of the above |  | 27 (1.8) | 40 (4.1) |  | 27 (1.8) | 40 (4.1) |  |
| Medical history: High cholesterol | 0 (0.0) | 220 (14.8) | 151 (15.7) | 0.544 | 220 (14.8) | 151 (15.7) | 0.544 |
| Medical history: Hypertension | 0 (0.0) | 310 (20.8) | 161 (16.7) | 0.012 | 310 (20.8) | 161 (16.7) | 0.012 |
| Medical history: Arthritis | 0 (0.0) | 166 (11.1) | 107 (11.1) | 0.975 | 166 (11.1) | 107 (11.1) | 0.975 |
| Medical history: Diabetes | 0 (0.0) | 349 (23.4) | 66 (6.8) | <0.001 | 349 (23.4) | 66 (6.8) | <0.001 |
| Medical history: Heart failure | 0 (0.0) | 98 (6.6) | 10 (1.0) | <0.001 | 98 (6.6) | 10 (1.0) | <0.001 |
| Medical history: Stroke | 0 (0.0) | 107 (7.2) | 5 (0.5) | <0.001 | 107 (7.2) | 5 (0.5) | <0.001 |
| Medical history: Bronchitis | 0 (0.0) | 77 (5.2) | 47 (4.9) | 0.747 | 77 (5.2) | 47 (4.9) | 0.747 |
| Medical history: Asthma | 0 (0.0) | 225 (15.1) | 90 (9.3) | <0.001 | 225 (15.1) | 90 (9.3) | <0.001 |
| Medical history: Depression | 0 (0.0) | 388 (26.0) | 186 (19.3) | <0.001 | 388 (26.0) | 186 (19.3) | <0.001 |
| Medical history: Migraine | 0 (0.0) | 134 (9.0) | 94 (9.8) | 0.528 | 134 (9.0) | 94 (9.8) | 0.528 |
| Medical history: Cancer | 0 (0.0) | 72 (4.8) | 38 (3.9) | 0.298 | 72 (4.8) | 38 (3.9) | 0.298 |
| Medical history: None | 0 (0.0) | 452 (30.3) | 403 (41.8) | <0.001 | 452 (30.3) | 403 (41.8) | <0.001 |
| Medical history: Other | 0 (0.0) | 64 (4.3) | 98 (10.2) | <0.001 | 64 (4.3) | 98 (10.2) | <0.001 |
| Smoking history | 2 (0.1) |  |  | <0.001 |  |  | <0.001 |
| Currently |  | 415 (27.9) | 139 (14.4) |  | 415 (27.9) | 139 (14.4) |  |
| In the past |  | 568 (38.1) | 262 (27.2) |  | 568 (38.1) | 262 (27.2) |  |
| Never |  | 506 (34.0) | 562 (58.4) |  | 507 (34.0) | 563 (58.4) |  |
| BMI category | 1 (0.0) |  |  | <0.001 |  |  | <0.001 |
| Underweight |  | 357 (24.0) | 35 (3.6) |  | 368 (24.7) | 45 (4.7) |  |
| Normal weight |  | 549 (36.8) | 395 (41.0) |  | 568 (38.1) | 408 (42.3) |  |
| Overweight |  | 320 (21.5) | 319 (33.1) |  | 330 (22.1) | 325 (33.7) |  |
| Obesity |  | 264 (17.7) | 214 (22.2) |  | 224 (15.0) | 186 (19.3) |  |
| Live alone | 1 (0.0) | 462 (31.0) | 236 (24.5) | <0.001 | 463 (31.1) | 236 (24.5) | <0.001 |
| Diagnosed with COVID-19 | 0 (0.0) | 384 (25.8) | 6 (0.6) | <0.001 | 384 (25.8) | 6 (0.6) | <0.001 |
| Experienced COVID-19-like symptoms not serious enough to require hospitalization | 0 (0.0) | 498 (33.4) | 94 (9.8) | <0.001 | 498 (33.4) | 94 (9.8) | <0.001 |
| Has a family member diagnosed with COVID-19 | 0 (0.0) | 440 (29.5) | 64 (6.6) | <0.001 | 440 (29.5) | 64 (6.6) | <0.001 |
| Knows someone with a COVID-19 diagnosis | 2 (0.1) | 730 (49.0) | 236 (24.5) | <0.001 | 730 (49.0) | 238 (24.7) | <0.001 |
| Under mandatory social distancing | 0 (0.0) | 1,117 (75.0) | 505 (52.4) | <0.001 | 1,117 (75.0) | 505 (52.4) | <0.001 |
| Practices social distancing personally | 0 (0.0) | 1,401 (94.0) | 901 (93.5) | 0.573 | 1,401 (94.0) | 901 (93.5) | 0.573 |
| Fear of COVID-19's impact on health (0-10 scale) | 0 (0.0) | 6.4 (2.6) | 5.0 (3.0) | <0.001 | 6.4 (2.6) | 5.0 (3.0) | <0.001 |
| Fear of COVID-19's impact on finances (0-10 scale) | 0 (0.0) | 6.6 (2.5) | 5.1 (3.2) | <0.001 | 6.6 (2.5) | 5.1 (3.2) | <0.001 |
| Supporting social distance policy (0-10 scale) | 0 (0.0) | 7.6 (2.2) | 7.3 (2.7) | <0.001 | 7.6 (2.2) | 7.3 (2.7) | <0.001 |
| Change of income due to COVID-19 | 0 (0.0) |  |  | <0.001 |  |  | <0.001 |
| Increased |  | 58 (3.9) | 34 (3.5) |  | 58 (3.9) | 34 (3.5) |  |
| Reduced |  | 980 (59.7) | 354 (36.7) |  | 980 (59.7) | 354 (36.7) |  |
| No change |  | 542 (36.4) | 576 (59.8) |  | 542 (36.4) | 576 (59.8) |  |
| Change of spending pattern | 0 (0.0) |  |  | <0.001 |  |  | <0.001 |
| More |  | 386 (25.9) | 125 (13.0) |  | 386 (25.9) | 125 (13.0) |  |
| Less |  | 766 (51.4) | 481 (49.9) |  | 766 (51.4) | 481 (49.9) |  |
| No change |  | 338 (22.7) | 358 (37.1) |  | 338 (22.7) | 358 (37.1) |  |
| Employment status | 0 (0.0) |  |  | <0.001 |  |  | <0.001 |
| Employed full-time |  | 1,129 (75.8) | 594 (61.6) |  | 1,129 (75.8) | 594 (61.6) |  |
| Employed part-time |  | 159 (10.7) | 143 (14.8) |  | 159 (10.7) | 143 (14.8) |  |
| Unemployed, seeking employment |  | 76 (5.1) | 52 (5.4) |  | 76 (5.1) | 52 (5.4) |  |
| Unemployed, not seeking employment |  | 30 (2.0) | 24 (2.5) |  | 30 (2.0) | 24 (2.5) |  |
| Student |  | 15 (1.0) | 10 (1.0) |  | 15 (1.0) | 10 (1.0) |  |
| Retired |  | 39 (2.6) | 92 (9.5) |  | 39 (2.6) | 92 (9.5) |  |
| On disability and can't work |  | 14 (0.9) | 14 (1.5) |  | 14 (0.9) | 14 (1.5) |  |
| Homemaker or stay at home parent |  | 24 (1.6) | 32 (3.3) |  | 24 (1.6) | 32 (3.3) |  |
| Don't know |  | 4 (0.3) | 3 (0.3) |  | 4 (0.3) | 3 (0.3) |  |
| Occupation | 429 (17.5) |  |  | <0.001 |  |  | <0.001 |
| Management |  | 246 (19.1) | 55 (7.5) |  | 272 (18.3) | 92 (9.5) |  |
| Business and Financial Operations |  | 272 (21.1) | 89 (12.1) |  | 308 (20.7) | 131 (13.6) |  |
| Computer and Mathematical |  | 218 (16.9) | 76 (10.3) |  | 249 (16.7) | 105 (10.9) |  |
| Architecture and Engineering |  | 49 (3.8) | 9 (1.2) |  | 58 (3.9) | 13 (1.3) |  |
| Life, Physical, and Social Science |  | 15 (1.2) | 19 (2.6) |  | 17 (1.1) | 22 (2.3) |  |
| Community and Social Service |  | 18 (1.4) | 12 (1.6) |  | 20 (1.3) | 14 (1.5) |  |
| Legal |  | 7 (0.5) | 9 (1.2) |  | 9 (0.6) | 11 (1.1) |  |
| Educational Instruction and Library |  | 64 (5.0) | 59 (8.0) |  | 81 (5.4) | 76 (7.9) |  |
| Arts, Design, Entertainment, Sports, and Media |  | 34 (2.6) | 48 (6.5) |  | 39 (2.6) | 55 (5.7) |  |
| Healthcare Practitioners and Technicians |  | 28 (2.2) | 23 (3.1) |  | 34 (2.3) | 31 (3.2) |  |
| Healthcare Support |  | 31 (2.4) | 34 (4.6) |  | 37 (2.5) | 39 (4.0) |  |
| Protective Service |  | 2 (0.2) | 5 (0.7) |  | 2 (0.1) | 5 (0.5) |  |
| Food Preparation and Serving |  | 23 (1.8) | 23 (3.1) |  | 28 (1.9) | 25 (2.6) |  |
| Building and Grounds Cleaning and Maintenance |  | 5 (0.4) | 8 (1.1) |  | 6 (0.4) | 10 (1.0) |  |
| Personal Care and Service |  | 15 (1.2) | 8 (1.1) |  | 20 (1.3) | 11 (1.1) |  |
| Sales |  | 71 (5.5) | 64 (8.7) |  | 89 (6.0) | 85 (8.8) |  |
| Office and Administrative Support |  | 70 (5.4) | 92 (12.5) |  | 80 (5.4) | 112 (11.6) |  |
| Farming, Fishing, and Forestry |  | 7 (0.5) | 7 (0.9) |  | 8 (0.5) | 8 (0.8) |  |
| Construction and Extraction |  | 11 (0.9) | 14 (1.9) |  | 17 (1.1) | 17 (1.8) |  |
| Installation, Maintenance, and Repair |  | 11 (0.9) | 10 (1.4) |  | 12 (0.8) | 10 (1.0) |  |
| Production and Manufacturing |  | 42 (3.3) | 25 (3.4) |  | 48 (3.2) | 32 (3.3) |  |
| Transportation and Material Moving |  | 21 (1.6) | 17 (2.3) |  | 23 (1.5) | 18 (1.9) |  |
| Military |  | 4 (0.3) | 3 (0.4) |  | 5 (0.3) | 5 (0.5) |  |
| Research |  | 14 (1.1) | 18 (2.4) |  | 16 (1.1) | 24 (2.5) |  |
| Don't know |  | 10 (0.8) | 10 (1.4) |  | 12 (0.8) | 13 (1.3) |  |
| Employment status change due to COVID-19 | 429 (17.5) |  |  | <0.001 |  |  | <0.001 |
| No change |  | 377 (29.3) | 398 (54.0) |  | 448 (30.1) | 491 (50.9) |  |
| Lost job |  | 142 (11.0) | 5 (0.7) |  | 158 (10.6) | 18 (1.9) |  |
| Work from home |  | 739 (57.4) | 294 (39.9) |  | 848 (56.9) | 403 (41.8) |  |
| Laid off temporarily |  | 30 (2.3) | 40 (5.4) |  | 36 (2.4) | 52 (5.4) |  |
| Work deemed essential? | 429 (17.5) |  |  | <0.001 |  |  | <0.001 |
| Yes |  | 768 (59.6) | 243 (33.0) |  | 869 (58.3) | 355 (36.8) |  |
| No |  | 458 (35.6) | 456 (61.9) |  | 552 (37.0) | 559 (58.0) |  |
| Don't know |  | 62 (4.8) | 38 (5.2) |  | 69 (4.6) | 50 (5.2) |  |
| Hours of work missed due to COVID-19 | 430 (17.5) | 6.1 (9.6) | 2.3 (7.9) | <0.001 | 5.8 (9.3) | 3.0 (8.5) | <0.001 |
| Hours of work missed not due to COVID-19 | 429 (17.5) | 4.8 (8.1) | 2.2 (6.0) | <0.001 | 4.5 (7.7) | 2.7 (6.7) | <0.001 |
| Average hours of work per day | 429 (17.5) | 6.6 (2.5) | 6.7 (2.6) | 0.157 | 6.6 (2.5) | 6.7 (2.6) | 0.331 |
| COVID-19 impact on productivity | 0 (0.0) | 5.5 (2.9) | 2.9 (2.5) | <0.001 | 5.5 (2.8) | 3.0 (2.4) | <0.001 |
| BRFSS Q1 Did not have enough food or money to get more | 0 (0.0) |  |  | <0.001 |  |  | <0.001 |
| Often true |  | 121 (8.1) | 19 (2.0) |  | 121 (8.1) | 19 (2.0) |  |
| Sometimes true |  | 633 (42.5) | 116 (12.0) |  | 633 (42.5) | 116 (12.0) |  |
| Never true |  | 683 (45.8) | 808 (83.8) |  | 683 (45.8) | 808 (83.8) |  |
| Don’t know/Not sure |  | 34 (2.3) | 19 (2.0) |  | 34 (2.3) | 19 (2.0) |  |
| Prefer not to answer |  | 19 (1.3) | 2 (0.2) |  | 19 (1.3) | 2 (0.2) |  |
| BRFSS Q2 Could not afford to eat balanced meal | 0 (0.0) |  |  | <0.001 |  |  | <0.001 |
| Often true |  | 205 (13.8) | 48 (5.0) |  | 205 (13.8) | 48 (5.0) |  |
| Sometimes true |  | 559 (37.5) | 150 (15.6) |  | 559 (37.5) | 150 (15.6) |  |
| Never true |  | 659 (44.2) | 748 (77.6) |  | 659 (44.2) | 748 (77.6) |  |
| Don’t know/Not sure |  | 53 (3.6) | 15 (1.6) |  | 53 (3.6) | 15 (1.6) |  |
| Prefer not to answer |  | 14 (0.9) | 3 (0.3) |  | 14 (0.9) | 3 (0.3) |  |
| BRFSS Q3 Finances at the end of the month | 0 (0.0) |  |  | <0.001 |  |  | <0.001 |
| End up with some money left over |  | 369 (24.8) | 491 (50.9) |  | 369 (24.8) | 491 (50.9) |  |
| Have just enough money to make ends meet |  | 846 (56.8) | 365 (37.9) |  | 846 (56.8) | 365 (37.9) |  |
| Not have enough money to make ends meet |  | 216 (14.5) | 90 (9.3) |  | 216 (14.5) | 90 (9.3) |  |
| Don’t know/ Not sure |  | 41 (2.8) | 11 (1.1) |  | 41 (2.8) | 11 (1.1) |  |
| Prefer not to answer |  | 18 (1.2) | 7 (0.7) |  | 18 (1.2) | 7 (0.7) |  |
| BRFSS Q4 Stress within the last 30 days | 0 (0.0) |  |  | <0.001 |  |  | <0.001 |
| None of the time |  | 128 (8.6) | 181 (18.8) |  | 128 (8.6) | 181 (18.8) |  |
| A little of the time |  | 479 (32.1) | 351 (36.4) |  | 479 (32.1) | 351 (36.4) |  |
| Some of the time |  | 574 (38.5) | 262 (27.2) |  | 574 (38.5) | 262 (27.2) |  |
| Most of the time |  | 240 (16.1) | 125 (13.0) |  | 240 (16.1) | 125 (13.0) |  |
| All of the time |  | 59 (4.0) | 41 (4.3) |  | 59 (4.0) | 41 (4.3) |  |
| Don’t know/ Not sure |  | 2 (0.1) | 3 (0.3) |  | 2 (0.1) | 3 (0.3) |  |
| Prefer not to answer |  | 8 (0.5) | 1 (0.1) |  | 8 (0.5) | 1 (0.1) |  |
| CD RISC Q1 Able to bounce back when changes occur | 0 (0.0) |  |  | <0.001 |  |  | <0.001 |
| Not true at all |  | 32 (2.1) | 30 (3.1) |  | 32 (2.1) | 30 (3.1) |  |
| Rarely true |  | 102 (6.8) | 70 (7.3) |  | 102 (6.8) | 70 (7.3) |  |
| Sometimes true |  | 454 (30.5) | 199 (20.6) |  | 454 (30.5) | 199 (20.6) |  |
| Often true |  | 675 (45.3) | 417 (43.3) |  | 675 (45.3) | 417 (43.3) |  |
| True nearly all the time |  | 227 (15.2) | 248 (25.7) |  | 227 (15.2) | 248 (25.7) |  |
| CD RISC Q2 Tend to bounce back after illness, injury, or other hardships | 0 (0.0) |  |  | <0.001 |  |  | <0.001 |
| Not true at all |  | 31 (2.1) | 32 (3.3) |  | 31 (2.1) | 32 (3.3) |  |
| Rarely true |  | 115 (7.7) | 63 (6.5) |  | 115 (7.7) | 63 (6.5) |  |
| Sometimes true |  | 446 (29.9) | 202 (21.0) |  | 446 (29.9) | 202 (21.0) |  |
| Often true |  | 599 (40.2) | 393 (40.8) |  | 599 (40.2) | 393 (40.8) |  |
| True nearly all the time |  | 299 (20.1) | 274 (28.4) |  | 299 (20.1) | 274 (28.4) |  |
| Have experienced traumatic events | 0 (0.0) | 813 (54.6) | 393 (40.8) | <0.001 | 813 (54.6) | 393 (40.8) | <0.001 |
| PROMIS Q1 Irritated more than people knew in the past seven days | 0 (0.0) |  |  | <0.001 |  |  | <0.001 |
| Never |  | 290 (19.5) | 388 (40.2) |  | 290 (19.5) | 388 (40.2) |  |
| Rarely |  | 293 (19.7) | 231 (24.0) |  | 293 (19.7) | 231 (24.0) |  |
| Sometimes |  | 460 (30.9) | 216 (22.4) |  | 460 (30.9) | 216 (22.4) |  |
| Often |  | 345 (23.2) | 94 (9.8) |  | 345 (23.2) | 94 (9.8) |  |
| Always |  | 102 (6.8) | 35 (3.6) |  | 102 (6.8) | 35 (3.6) |  |
| PROMIS Q2 Felt angry in the past seven days | 0 (0.0) |  |  | <0.001 |  |  | <0.001 |
| Never |  | 329 (22.1) | 450 (46.7) |  | 329 (22.1) | 450 (46.7) |  |
| Rarely |  | 324 (21.7) | 254 (26.3) |  | 324 (21.7) | 254 (26.3) |  |
| Sometimes |  | 456 (30.6) | 180 (18.7) |  | 456 (30.6) | 180 (18.7) |  |
| Often |  | 284 (19.1) | 60 (6.2) |  | 284 (19.1) | 60 (6.2) |  |
| Always |  | 97 (6.5) | 20 (2.1) |  | 97 (6.5) | 20 (2.1) |  |
| PROMIS Q3 Felt like I was ready to explode in the past seven days | 0 (0.0) |  |  | <0.001 |  |  | <0.001 |
| Never |  | 438 (29.4) | 653 (67.7) |  | 438 (29.4) | 653 (67.7) |  |
| Rarely |  | 266 (17.9) | 167 (17.3) |  | 266 (17.9) | 167 (17.3) |  |
| Sometimes |  | 332 (22.3) | 91 (9.4) |  | 332 (22.3) | 91 (9.4) |  |
| Often |  | 322 (21.6) | 36 (3.7) |  | 322 (21.6) | 36 (3.7) |  |
| Always |  | 132 (8.9) | 17 (1.8) |  | 132 (8.9) | 17 (1.8) |  |
| PROMIS Q4 Was grouchy in the past seven days | 0 (0.0) |  |  | <0.001 |  |  | <0.001 |
| Never |  | 278 (18.7) | 369 (38.3) |  | 278 (18.7) | 369 (38.3) |  |
| Rarely |  | 274 (18.4) | 284 (29.5) |  | 274 (18.4) | 284 (29.5) |  |
| Sometimes |  | 423 (28.4) | 205 (21.3) |  | 423 (28.4) | 205 (21.3) |  |
| Often |  | 352 (23.6) | 77 (8.0) |  | 352 (23.6) | 77 (8.0) |  |
| Always |  | 163 (10.9) | 29 (3.0) |  | 163 (10.9) | 29 (3.0) |  |
| PROMIS Q5 Felt annoyed in the past seven days | 0 (0.0) |  |  | <0.001 |  |  | <0.001 |
| Never |  | 225 (15.1) | 285 (29.6) |  | 225 (15.1) | 285 (29.6) |  |
| Rarely |  | 285 (19.1) | 262 (27.2) |  | 285 (19.1) | 262 (27.2) |  |
| Sometimes |  | 397 (26.6) | 272 (28.2) |  | 397 (26.6) | 272 (28.2) |  |
| Often |  | 413 (27.7) | 106 (11.0) |  | 413 (27.7) | 106 (11.0) |  |
| Always |  | 170 (11.4) | 39 (4.0) |  | 170 (11.4) | 39 (4.0) |  |
| PHQ-4 score | 0 (0.0) |  |  | <0.001 |  |  | <0.001 |
| 0-2 |  | 404 (27.1) | 565 (58.6) |  | 404 (27.1) | 565 (58.6) |  |
| 3-5 |  | 264 (17.7) | 197 (20.4) |  | 264 (17.7) | 197 (20.4) |  |
| 6-8 |  | 594 (39.9) | 126 (13.1) |  | 594 (39.9) | 126 (13.1) |  |
| 9-12 |  | 228 (15.3) | 76 (7.9) |  | 228 (15.3) | 76 (7.9) |  |
| EQ-5D-5L Mobility | 3 (0.1) |  |  | <0.001 |  |  | <0.001 |
| No problems walking |  | 790 (53.1) | 802 (83.4) |  | 790 (53.0) | 803 (83.3) |  |
| Slight problems walking |  | 317 (21.3) | 101 (10.5) |  | 317 (21.3) | 101 (10.5) |  |
| Moderate problems walking |  | 299 (20.1) | 47 (4.9) |  | 300 (20.1) | 48 (5.0) |  |
| Severe problems walking |  | 67 (4.5) | 7 (0.7) |  | 67 (4.5) | 7 (0.7) |  |
| Unable to walk |  | 16 (1.1) | 5 (0.5) |  | 16 (1.1) | 5 (0.5) |  |
| EQ-5D-5L Self-care | 3 (0.1) |  |  | <0.001 |  |  | <0.001 |
| No problems washing or dressing myself |  | 857 (57.6) | 886 (92.0) |  | 857 (57.5) | 887 (92.0) |  |
| Slight problems washing or dressing myself |  | 273 (18.3) | 53 (5.5) |  | 273 (18.3) | 53 (5.5) |  |
| Moderate problems washing or dressing myself |  | 263 (17.7) | 16 (1.7) |  | 265 (17.8) | 16 (1.7) |  |
| Severe problems washing or dressing myself |  | 82 (5.5) | 7 (0.7) |  | 82 (5.5) | 7 (0.7) |  |
| Unable to wash or dress myself |  | 13 (0.9) | 1 (0.1) |  | 13 (0.9) | 1 (0.1) |  |
| EQ-5D-5L Usual activities | 0 (0.0) |  |  | <0.001 |  |  | <0.001 |
| No problems doing usual activities |  | 744 (49.9) | 766 (79.5) |  | 744 (49.9) | 766 (79.5) |  |
| Slight problems doing usual activities |  | 387 (26.0) | 136 (14.1) |  | 387 (26.0) | 136 (14.1) |  |
| Moderate problems doing usual activities |  | 257 (17.2) | 47 (4.9) |  | 257 (17.2) | 47 (4.9) |  |
| Severe problems doing usual activities |  | 95 (6.4) | 13 (1.3) |  | 95 (6.4) | 13 (1.3) |  |
| Unable to do usual activities |  | 7 (0.5) | 2 (0.2) |  | 7 (0.5) | 2 (0.2) |  |
| EQ-5D-5L Pain/Discomfort | 3 (0.1) |  |  | <0.001 |  |  | <0.001 |
| No pain or discomfort |  | 562 (37.7) | 487 (50.6) |  | 562 (37.7) | 487 (50.5) |  |
| Slight pain or discomfort |  | 492 (33.0) | 318 (33.1) |  | 492 (33.0) | 319 (33.1) |  |
| Moderate or discomfort |  | 308 (20.7) | 127 (13.2) |  | 309 (20.7) | 128 (13.3) |  |
| Severe pain or discomfort |  | 108 (7.3) | 23 (2.4) |  | 108 (7.2) | 23 (2.4) |  |
| Extreme pain or discomfort |  | 19 (1.3) | 7 (0.7) |  | 19 (1.3) | 7 (0.7) |  |
| EQ-5D-5L Anxiety/Depression | 0 (0.0) |  |  | <0.001 |  |  | <0.001 |
| Not anxious or depressed |  | 445 (29.9) | 456 (47.3) |  | 445 (29.9) | 456 (47.3) |  |
| Slightly anxious or depressed |  | 512 (34.4) | 270 (28.0) |  | 512 (34.4) | 270 (28.0) |  |
| Moderately anxious or depressed |  | 361 (24.2) | 163 (16.9) |  | 361 (24.2) | 163 (16.9) |  |
| Severely anxious or depressed |  | 141 (9.5) | 52 (5.4) |  | 141 (9.5) | 52 (5.4) |  |
| Extremely anxious or depressed |  | 31 (2.1) | 23 (2.4) |  | 31 (2.1) | 23 (2.4) |  |
| EQ-5D-5L VAS | 0 (0.0) | 80.1 (16.2) | 76.4 (19.3) | <0.001 | 80.1 (16.2) | 76.4 (19.3) | <0.001 |
| EQ-5D-5L utility | 7 (0.3) | 0.7 (0.3) | 0.9 (0.2) | <0.001 | 0.7 (0.3) | 0.8 (0.2) | <0.001 |
| VR12 Q1 General Health status | 1 (0.0) |  |  | <0.001 |  |  | <0.001 |
| Excellent |  | 230 (15.4) | 115 (11.9) |  | 230 (15.4) | 115 (11.9) |  |
| Very good |  | 562 (37.7) | 368 (38.2) |  | 562 (37.7) | 368 (38.2) |  |
| Good |  | 556 (37.3) | 316 (32.8) |  | 556 (37.3) | 316 (32.8) |  |
| Fair |  | 127 (8.5) | 150 (15.6) |  | 127 (8.5) | 151 (15.7) |  |
| Poor |  | 15 (1.0) | 14 (1.5) |  | 15 (1.0) | 14 (1.5) |  |
| VR12 Q2a Limit in moderate activities | 2 (0.1) |  |  | <0.001 |  |  | <0.001 |
| Limited a lot |  | 180 (12.1) | 40 (4.2) |  | 180 (12.1) | 40 (4.1) |  |
| Limited a little |  | 718 (48.2) | 137 (14.2) |  | 718 (48.2) | 137 (14.2) |  |
| Not limited at all |  | 591 (39.7) | 786 (81.6) |  | 592 (39.7) | 787 (81.6) |  |
| VR12 Q2b Limit in climbing several flights of stairs | 2 (0.1) |  |  | <0.001 |  |  | <0.001 |
| Limited a lot |  | 180 (12.1) | 40 (4.2) |  | 180 (12.1) | 40 (4.1) |  |
| Limited a little |  | 718 (48.2) | 137 (14.2) |  | 718 (48.2) | 137 (14.2) |  |
| Not limited at all |  | 591 (39.7) | 786 (81.6) |  | 592 (39.7) | 787 (81.6) |  |
| VR12 Q3a Accomplished less due to physical health | 1 (0.0) |  |  | <0.001 |  |  | <0.001 |
| None of the time |  | 389 (26.1) | 612 (63.5) |  | 389 (26.1) | 612 (63.5) |  |
| A little of the time |  | 273 (18.3) | 176 (18.3) |  | 273 (18.3) | 176 (18.3) |  |
| Some of the time |  | 451 (30.3) | 104 (10.8) |  | 451 (30.3) | 104 (10.8) |  |
| Most of the time |  | 289 (19.4) | 48 (5.0) |  | 290 (19.5) | 48 (5.0) |  |
| All of the time |  | 87 (5.8) | 24 (2.5) |  | 87 (5.8) | 24 (2.5) |  |
| VR12 Q3b Limited in work/activities due to physical health | 0 (0.0) |  |  | <0.001 |  |  | <0.001 |
| None of the time |  | 425 (28.5) | 671 (69.6) |  | 425 (28.5) | 671 (69.6) |  |
| A little of the time |  | 235 (15.8) | 145 (15.0) |  | 235 (15.8) | 145 (15.0) |  |
| Some of the time |  | 383 (25.7) | 79 (8.2) |  | 383 (25.7) | 79 (8.2) |  |
| Most of the time |  | 347 (23.3) | 44 (4.6) |  | 347 (23.3) | 44 (4.6) |  |
| All of the time |  | 100 (6.7) | 25 (2.6) |  | 100 (6.7) | 25 (2.6) |  |
| VR12 Q4a Accomplished less due to emotional problems | 1 (0.0) |  |  | <0.001 |  |  | <0.001 |
| None of the time |  | 320 (21.5) | 493 (51.1) |  | 320 (21.5) | 493 (51.1) |  |
| A little of the time |  | 283 (19.0) | 227 (23.5) |  | 283 (19.0) | 227 (23.5) |  |
| Some of the time |  | 451 (30.3) | 154 (16.0) |  | 451 (30.3) | 154 (16.0) |  |
| Most of the time |  | 352 (23.6) | 65 (6.7) |  | 353 (23.7) | 65 (6.7) |  |
| All of the time |  | 83 (5.6) | 25 (2.6) |  | 83 (5.6) | 25 (2.6) |  |
| VR12 Q4b Didn’t do work or other activities as carefully due to emotional problems | 0 (0.0) |  |  | <0.001 |  |  | <0.001 |
| None of the time |  | 381 (25.6) | 577 (59.9) |  | 381 (25.6) | 577 (59.9) |  |
| A little of the time |  | 293 (19.7) | 221 (22.9) |  | 293 (19.7) | 221 (22.9) |  |
| Some of the time |  | 397 (26.6) | 120 (12.4) |  | 397 (26.6) | 120 (12.4) |  |
| Most of the time |  | 327 (21.9) | 29 (3.0) |  | 327 (21.9) | 29 (3.0) |  |
| All of the time |  | 92 (6.2) | 17 (1.8) |  | 92 (6.2) | 17 (1.8) |  |
| VR12 Q5 Pain interfere with normal work | 6 (0.2) |  |  | <0.001 |  |  | <0.001 |
| Not at all |  | 410 (27.6) | 556 (57.9) |  | 410 (27.5) | 557 (57.8) |  |
| A little bit |  | 472 (31.7) | 267 (27.8) |  | 473 (31.7) | 269 (27.9) |  |
| Moderately |  | 450 (30.3) | 93 (9.7) |  | 451 (30.3) | 93 (9.6) |  |
| Quite a bit |  | 99 (6.7) | 36 (3.7) |  | 100 (6.7) | 36 (3.7) |  |
| Extremely |  | 56 (3.8) | 9 (0.9) |  | 56 (3.8) | 9 (0.9) |  |
| VR12 Q6a Felt calm and peaceful | 0 (0.0) |  |  | <0.001 |  |  | <0.001 |
| All of the time |  | 100 (6.7) | 94 (9.8) |  | 100 (6.7) | 94 (9.8) |  |
| Most of the time |  | 297 (19.9) | 337 (35.0) |  | 297 (19.9) | 337 (35.0) |  |
| A good bit of the time |  | 297 (19.9) | 165 (17.1) |  | 297 (19.9) | 165 (17.1) |  |
| Some of the time |  | 418 (28.1) | 172 (17.8) |  | 418 (28.1) | 172 (17.8) |  |
| A little of the time |  | 299 (20.1) | 144 (14.9) |  | 299 (20.1) | 144 (14.9) |  |
| None of the time |  | 79 (5.3) | 52 (5.4) |  | 79 (5.3) | 52 (5.4) |  |
| VR12 Q6b Have a lot of energy | 1 (0.0) |  |  | <0.001 |  |  | <0.001 |
| All of the time |  | 79 (5.3) | 59 (6.1) |  | 79 (5.3) | 59 (6.1) |  |
| Most of the time |  | 239 (16.0) | 209 (21.7) |  | 239 (16.0) | 209 (21.7) |  |
| A good bit of the time |  | 292 (19.6) | 232 (24.1) |  | 292 (19.6) | 232 (24.1) |  |
| Some of the time |  | 428 (28.7) | 205 (21.3) |  | 428 (28.7) | 205 (21.3) |  |
| A little of the time |  | 334 (22.4) | 168 (17.4) |  | 334 (22.4) | 168 (17.4) |  |
| None of the time |  | 118 (7.9) | 90 (9.3) |  | 118 (7.9) | 91 (9.4) |  |
| VR12 Q6c Felt downhearted and blue | 0 (0.0) |  |  | <0.001 |  |  | <0.001 |
| All of the time |  | 53 (3.6) | 36 (3.7) |  | 53 (3.6) | 36 (3.7) |  |
| Most of the time |  | 97 (6.5) | 76 (7.9) |  | 97 (6.5) | 76 (7.9) |  |
| A good bit of the time |  | 217 (14.6) | 97 (10.1) |  | 217 (14.6) | 97 (10.1) |  |
| Some of the time |  | 417 (28.0) | 154 (16.0) |  | 417 (28.0) | 154 (16.0) |  |
| A little of the time |  | 449 (30.1) | 291 (30.2) |  | 449 (30.1) | 291 (30.2) |  |
| None of the time |  | 257 (17.2) | 310 (32.2) |  | 257 (17.2) | 310 (32.2) |  |
| VR12 Q7 Physical health or emotional problems interfered with social activities | 9 (0.4) |  |  | <0.001 |  |  | <0.001 |
| All of the time |  | 86 (5.8) | 25 (2.6) |  | 86 (5.8) | 25 (2.6) |  |
| Most of the time |  | 329 (22.1) | 58 (6.0) |  | 329 (22.1) | 58 (6.0) |  |
| Some of the time |  | 497 (33.4) | 127 (13.2) |  | 498 (33.4) | 129 (13.4) |  |
| A little of the time |  | 250 (16.8) | 209 (21.8) |  | 251 (16.8) | 212 (22.0) |  |
| None of the time |  | 324 (21.8) | 540 (56.3) |  | 326 (21.9) | 540 (56.0) |  |
| VR12 Q8 Physical health compared to one year ago | 1 (0.0) |  |  | <0.001 |  |  | <0.001 |
| Much better |  | 193 (13.0) | 54 (5.6) |  | 193 (13.0) | 54 (5.6) |  |
| Slightly better |  | 436 (29.3) | 137 (14.2) |  | 436 (29.3) | 137 (14.2) |  |
| About the same |  | 664 (44.6) | 582 (60.4) |  | 664 (44.6) | 582 (60.4) |  |
| Slightly worse |  | 178 (11.9) | 172 (17.9) |  | 178 (11.9) | 173 (17.9) |  |
| Much worse |  | 19 (1.3) | 18 (1.9) |  | 19 (1.3) | 18 (1.9) |  |
| VR12 Q9 Emotional problems compared to one year ago | 1 (0.0) |  |  | <0.001 |  |  | <0.001 |
| Much better |  | 183 (12.3) | 55 (5.7) |  | 183 (12.3) | 55 (5.7) |  |
| Slightly better |  | 419 (28.1) | 121 (12.6) |  | 419 (28.1) | 121 (12.6) |  |
| About the same |  | 582 (39.1) | 460 (47.8) |  | 582 (39.1) | 460 (47.7) |  |
| Slightly worse |  | 260 (17.4) | 240 (24.9) |  | 260 (17.4) | 241 (25.0) |  |
| Much worse |  | 46 (3.1) | 87 (9.0) |  | 46 (3.1) | 87 (9.0) |  |
| Self-rated survey difficulty (0-10 scale) | 0 (0.0) | 4.2 (3.4) | 1.0 (1.7) | <0.001 | 4.2 (3.4) | 1.0 (1.7) | <0.001 |
| Self-rated survey usefulness (0-10 scale) | 0 (0.0) | 7.0 (2.0) | 6.3 (2.3) | <0.001 | 7.0 (2.0) | 6.3 (2.3) | <0.001 |

Note: Due to outlier removal, differences may exist before and after processing even if there was no missing in the variable.
